# Predicting continuous load-settlement trajectories of bored piles: A GA-optimized weighted ensemble framework with explainable AI

**DOI:** 10.1371/journal.pone.0360945

**Published:** 2026-10-21

**Authors:** Loi Van Giap, Tuong Lai Nguyen, Tuan Anh Pham

**Affiliations:** 1 University of Transport Technology, Hanoi, Vietnam; 2 Le Quy Don Technical University, Hanoi, Vietnam; Hohai University, CHINA

## Abstract

Accurate prediction of bored pile settlement is a core element in reliable foundation design. While machine learning (ML) models demonstrate superior predictive capabilities compared to traditional analytical methods, their black-box nature and tendency to generate non-physical trajectories significantly hinder their practical application in geotechnical engineering. This study proposes a new computational framework that ensures physical consistency by combining Weighted Ensemble learning with SHAP (SHapley Additive exPlanations) interpretation capabilities. The integrated structure incorporates linear regression (LR), Random Forest (RF), XGBoost, and multilayer neural networks (MLP) to capture the complex interaction between piles and soil through a database of 563 field static load test records. A two-stage hybrid optimization strategy, using Random Search combined with a Genetic Algorithm (GA), was implemented to optimize the global weight set. The results showed that the GA-Ensemble model achieved excellent accuracy on the independent test set (R^2^ = 0.958, NRMSE = 5.27%, RMSE = 0.380, MAE = 0.2322), successfully neutralizing the ladder errors of the tree model to recreate a smooth, continuous, and mechanically compliant load-settlement (P-S) trajectory. Notably, by converting the SHAP value to a real physical scale (millimeters), the study clarified the algorithm’s inference logic. SHAP dependency analysis demonstrated that the model successfully captures the coupling characteristics of elastic compression and geometric nonlinearity by identifying the interaction between external force (P) and free length (Lf). Local diagnostic decompositions precisely extract failure mechanisms, successfully quantifying the “systemic penalty” caused by elastic shortening of structures and foundation puncture even in very stiff soil layers. In short, the proposed method has gone beyond the limits of a purely interpolation tool, establishing a transparent and geomechanically reliable expert system for predicting settlement.

## 1. Introduction

Foundation design is one of the most crucial stages in construction engineering, and bored piles are a popular solution for high-rise buildings and transportation infrastructure due to their high load-bearing capacity. Accurately predicting pile head settlement under axial load not only ensures structural safety but also helps optimize project costs [1,2]. Typically, to accurately determine the load-settlement relationship at the pile head, static pile load tests are conducted in the field [3–6]. However, such pile load tests are often expensive and time-consuming, making them difficult to implement on a large scale [7–9]. Therefore, developing reliable settlement prediction tools as a preliminary design solution before actual load testing is an urgent requirement.

It must be acknowledged that predicting foundation settlement remains a classic challenge for geotechnical engineers. The behavior of bored piles is highly nonlinear, governed by the complex interaction mechanisms between the pile structure and the surrounding multilayered soil [10–12]. The heterogeneous variation of soil mechanical parameters (deformation modulus, penetration resistance, cohesion), errors in geological surveys, and geometric factors of the piles create significant obstacles [13–15]. In practice, theoretical methods based on analytical formulas often yield large errors due to oversimplification, assuming the soil is a homogeneous or purely linearly elastic medium [16]. On the other hand, numerical simulation methods such as finite element modeling (FEM), although powerful, are extremely sensitive to input parameters. The results of FEM depend heavily on the soil constitutive model, the type of contact element, and boundary conditions [17], requiring complex correction processes to converge with the actual load-displacement curve [18,19].

With the explosion of data science, Machine Learning (ML) models have demonstrated superior capabilities in establishing nonlinear mapping functions between input features (pile size, soil parameters, load) and output (settlement). Artificial Neural Network (ANN) models are pioneering tools in this field; for example, the study by Shahin et al. (2002) [20]demonstrated that ANNs are superior to traditional formulas. Recently, Kim et al. (2022) [21] and Zang et al. (2020) [22] successfully predicted settlement due to urban tunnel construction using several machine learning models combined with optimization algorithms. Khajehzadeh et al. (2024) [23] developed several efficient hybrid machine learning algorithms to estimate the settlement of shallow foundations on non-cohesive soils. Sharma et al. (2025) [24] successfully developed an artificial neural network to predict the settlement of embankments built on weak soil. Zhang et al. (2025) [25] developed a series of machine learning models to predict the settlement of shallow foundations based on SPT results, and also used Monte-Carlo simulations to assess the model uncertainty, considering that the training data was randomly split. Kirts et al. (2019) [26] published a model for determining soil consolidation settlement based on a machine-assisted vector model, with input variables being compression and recompression indices; the predicted consolidation settlement results were equivalent to the measured experimental results. Shuman et al. (2024) [27] developed a series of machine learning models, based on the basic parameters of piles and soil, to predict the settlement of large-diameter helical piles under total load. Although the data used for the new model was only extracted from FEM simulations, the highly accurate estimation of pile settlement under load also showed the great potential of ML models. El Gendy et al. (2025) [28] successfully developed a series of independent machine learning models to predict the load-settlement relationship of pile tops, showing the great potential of ML in handling complex geotechnical problems. However, most current studies still reveal systemic limitations. Firstly, the application of standalone machine learning models often leads to instability. A model may achieve very high performance on the training set but suffer serious degradation in accuracy on new data (overfitting) [29–31].

To address this problem, Ensemble Learning has emerged as a groundbreaking solution. Instead of relying on a single algorithm, the Weighted Ensemble voting model combines the advantages of multiple underlying models (ANN, RF, XGBoost) [32–35]. The studies by Bello et al. (2025) [36] built a machine learning model to predict pile settlement based on the CPT criterion. The input parameters were selected by voting based on a genetic algorithm. In addition, Chen et al. (2024) [37] developed an ensemble machine learning model, based on a weighted majority voting technique, to estimate slope stability. The results showed that the ensemble machine learning model performed better than the baseline models. Pham et al. (2021) [38] compared and combined machine learning models such as Random Forest, Gradient Boosting Machine, and ANN in the problem of predicting the axial bearing capacity of piles. The authors asserted that the weighted combination model helps to take advantage of the strengths of tree-based algorithms (in handling geological data noise) and ANN (in capturing nonlinearity), thereby providing more stable prediction results on many different soil types. Wang et al.[39] used stacking ensemble techniques to successfully build an ensemble machine learning model for predicting the boundaries between soil layers. Similarly, Shan Lin et al.[40] developed a stacked ensemble machine learning model for geotechnical stability analysis. The research results showed that the combinatorial learning model performed significantly better than the baseline models. Overall, studies have shown that when the weights of each component in the model are scientifically optimized instead of simply averaged, the model can predict pile head settlement with extremely high reliability even under limited input data. Furthermore, combined machine learning models consistently yield more impressive results and generalize data better than basic models.

Secondly, and perhaps most significantly, the research gap is the black-box nature of ML models and their tendency to generate non-physical predictions [41]. In geotechnical engineering, the load-settlement (P-S) curve must follow a monotonically increasing and continuous pattern. The application of decision tree-based models (such as Random Forest, XGBoost) often produces step-wise discontinuities or discrete interpolation in prediction curves [42,43], which does not accurately reflect the phase transition from elastic to plastic of the pile-soil system [44]. Furthermore, the inability of engineers to explain the internal logic behind ML’s prediction figures makes this tool difficult to accept in foundation design standards that demand absolute reliability and transparency [45].

To thoroughly overcome these barriers, this study proposes an advanced computational framework: an optimized weighted ensemble learning (GA) model combined with an explainable AI (SHAP) platform. The core objective is to establish an expert system that not only boasts excellent out-of-sample prediction accuracy but also has the ability to smoothly, continuously, and consistently redraw the load-settlement (P-S) trajectory in accordance with mechanical laws.

The groundbreaking contributions of this paper include:

- Developing a two-stage hybrid optimization strategy (Random Search combined with Genetic Algorithm): Automatically searching for and allocating optimal weights to heterogeneous composite structures (LR, RF, XGBoost, MLP), eliminating variance and completely overcoming the phenomenon of jagged predictions, thereby recreating a continuous P-S curve and adhering to thermodynamic principles.
- Demystifying the Black Box mechanism in real physical space: Pioneering the integration of SHAP analysis and direct conversion to millimeter (mm) coefficients, transforming AI from a statistical tool into a transparent geomechanical diagnostic system.
- Exploring the algorithm’s self-learning capabilities in physics: For the first time, quantitatively demonstrating that a machine learning platform can automatically discover and adhere to Hooke’s law of elasticity, identify P-Delta effects and foundation puncture mechanisms through local SHAP decomposition in extreme load scenarios.

## 2. Research methods

### 2.1. Research framework

To overcome the inherent limitations of standalone machine learning architectures and the pervasive black-box nature of data-driven models, this study proposes a holistic, physics-consistent computational framework. The overall architecture of the proposed Genetic Algorithm-Optimized Weighted Ensemble Learning coupled with SHAP interpretability is schematically illustrated in Fig 1. The framework is systematically structured into five interlinked phases:

**Fig 1:**
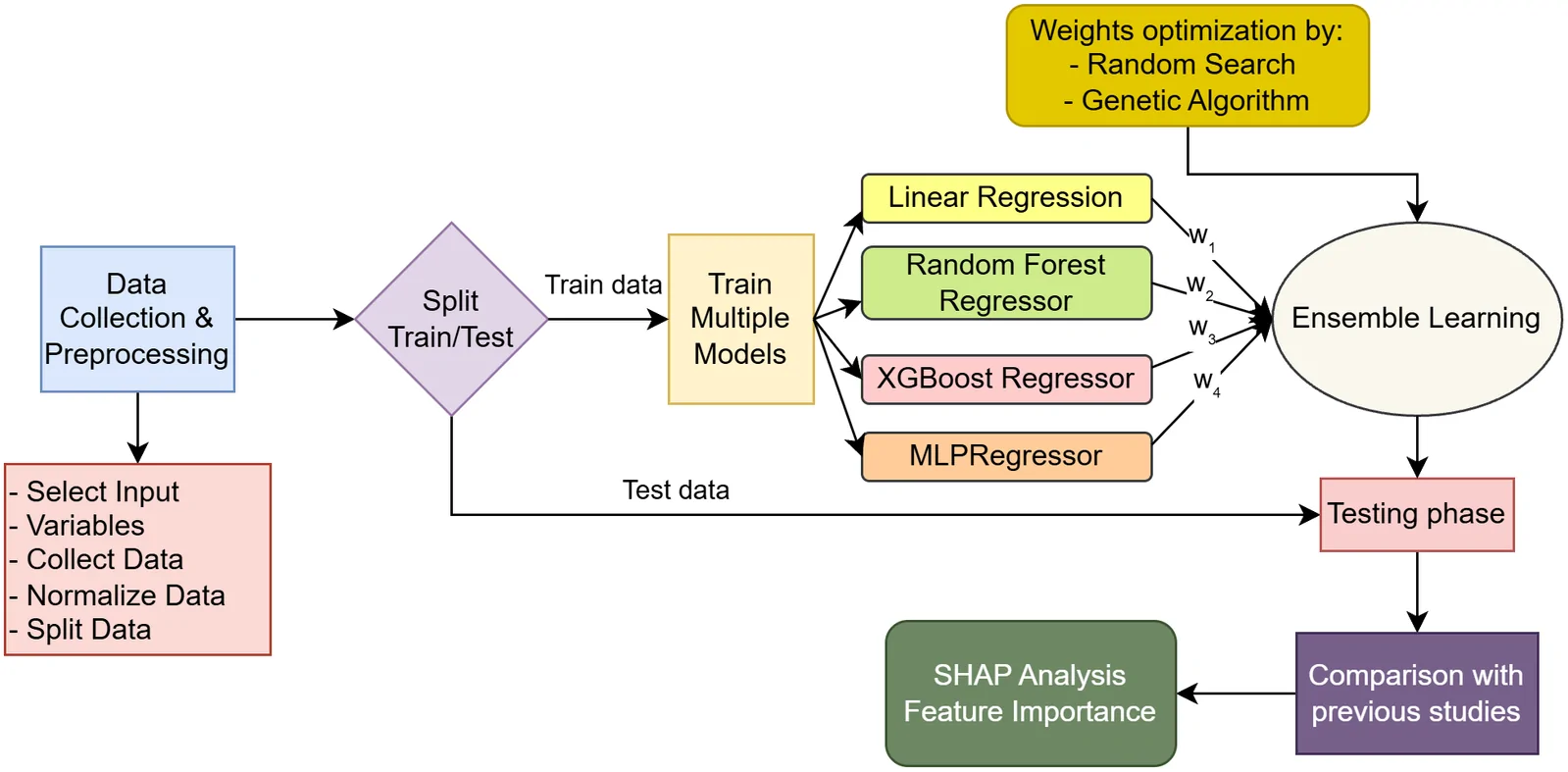
Schematic flowchart of the proposed GA-optimized Weighted Ensemble framework with SHAP interpretability.

- Phase 1: Data Curation and Strict Isolation. The pipeline initiates with the collection and preprocessing of the geotechnical variables. To ensure equitable contribution to the loss function and accelerate mathematical convergence, the raw input features are normalized. Crucially, to completely preclude the risk of data leakage, a common methodological flaw in applied ML studies, the dataset is strictly partitioned into a Training Set and an Independent Testing Set. The testing data is rigorously quarantined and strictly unseen during the model training and weight optimization phases.
- Phase 2: Heterogeneous Base Model Construction. Rather than relying on a single algorithmic paradigm, the framework deploys a heterogeneous suite of base regressors on the training data. This includes Linear Regression (LR) to establish a linear baseline, Random Forest (RF) for variance reduction via bagging, XGBoost for sequential bias minimization, and a Multi-Layer Perceptron (MLP) for continuous global function approximation. This algorithmic diversity ensures that both local geotechnical anomalies and global non-linear consolidation trends are captured.
- Phase 3: Two-Stage Hybrid Weight Optimization. This is the core engine of the proposed methodology. Instead of applying empirical or simple average voting, the ensemble weights (w_1_, w_2_, w_3_, w_4_) are treated as a constrained, non-convex optimization problem. A hybrid strategy is executed: Random Search is first deployed for coarse-grained global exploration to identify high-affinity hyper-simplex zones, followed immediately by a Genetic Algorithm (GA) for fine-grained evolutionary exploitation. This ensures the weight vector converges to the absolute global minimum of the validation error.
- Phase 4: Out-of-Sample Validation and Benchmarking. The locked GA-weighted ensemble model is subsequently exposed to the quarantined Testing phase. The predictive performance is comprehensively evaluated using statistical error metrics and then rigorously compared with previous studies to establish its out-of-sample generalization superiority.
- Phase 5: Geomechanical Interpretability via SHAP. In the final phase, the optimized ensemble is subjected to SHapley Additive exPlanations (SHAP) analysis. This Explainable AI (XAI) module systematically deconstructs the ensemble’s predictions, transitioning importance and dependence plots are utilized to extract the underlying physical laws and verify the model’s geomechanical consistency.

### 2.2. Data collection and description

The dataset utilized in this study was aggregated from two primary sources, comprising high-fidelity, full-scale Static Load Test (SLT) records for bored piles. The first subset consists of 47 international test scenarios extracted from the Federal Highway Administration (FHWA) database [46]. This validated, standard repository encompasses a highly diverse range of soil stratigraphies and construction conditions. The second subset incorporates 4 actual field SLT records from major infrastructure projects in Vietnam. This dataset includes full-scale static load tests performed on four bored piles located in Ho Chi Minh City, Vietnam. The field testing was executed in strict accordance with the Vietnamese Standard TCVN 9393:2012. The integration of these local cases enables the computational model to capture specific regional geological signatures – such as coastal soft soils and alluvial clays – thereby enhancing the framework’s practical applicability in local contexts. In total, the compiled database encompasses 51 distinct pile test scenarios (Table 1), yielding 542 discrete load-settlement data points (S1 File). This comprehensive dataset provides a sufficiently broad and heterogeneous feature space for the rigorous training and validation of the proposed machine learning architectures.

**Table 1:** Data used for the study.

Location	No. of Piles	Pile Type	Project Name
Phoenix, USA	10	Bored pile	Phoenix, AZ, Site C
Phoenix, USA	8	Bored pile	Phoenix, AZ, Site B
Phoenix, USA	7	Bored pile	Phoenix, AZ, Site A
Denver, USA	7	Bored pile	Denver Int. Airport, Site # 1
Florida, USA	1	Bored pile	171 SKYWAY Bridge, Site # 1
West Palm Beach	2	Bored pile	183 WEST PALM BEACH ADMN OFF
Jacksonville	1	Bored pile	193 BARNETT CENTER, JACKSONVILLE
Miami	1	Bored pile	195 S.E. FINANCIAL CENTER, MIAMI
Tampa	1	Bored pile	196 TAMPA INTERNATIONAL AIRPORT-2
Tampa	1	Bored pile	197 MEMORIAL HOSPITAL, TAMPA
Jacksonville	3	Bored pile	198 S.E. BANK BUILDING, JAX
Port Orange	1	Bored pile	200 PORT ORANGE BRIDGE, FL
Dade County	1	Bored pile	215 RADIO TOWER, DADE COUNTY, FL
Port Orange	1	Bored pile	216 PORT ORANGE BRIDGE, FL SHAFT3
Tampa	1	Bored pile	218 AMERICANA HOTEL – TAMPA
Tallahassee	1	Bored pile	219 LEWIS STATE BANK – TALLAHASSEE
Ho Chi Minh City, Vietnam	2	Bored pile	Kenton Project Complex
Ho Chi Minh City, Vietnam	2	Bored pile	Ben Van Don Apartment

To predict the pile head settlement (S), this study identified seven input parameters representing pile geometry, material properties, geological conditions, and applied loads. Unlike purely data-driven models that rely on arbitrary or scattered feature selection, the input variables in this study were strictly guided by foundation mechanics principles, ensuring a comprehensive representation of the load-transfer and deformation mechanisms within the pile-soil system through four distinct mechanistic groups.

The first group, Mechanical Driving Force, consists solely of the applied load (P), which serves as the primary independent variable and active external force exerted on the pile head. Within the non-linear response domain, the variation of P directly dictates whether the pile-soil system operates in the elastic phase or transitions toward the ultimate plastic yielding state.

The second group, Geometric Boundary Conditions and Spatial Interaction, incorporates three main parameters. These include the Pile Diameter (D), which simultaneously governs the cross-sectional area determining stress dissipation capacity and compressive resistance, alongside the shaft perimeter defining the contact area available to mobilize skin friction. It also features the Embedded Pile Length (Ls), representing the effective interaction depth between the pile shaft and surrounding soil to directly quantify the total surface area capable of generating shear stresses for ultimate shaft resistance, and the Free-Standing Pile Length ($L_{f}$). This unconfined pile section provides zero lateral frictional resistance and represents the zone most severely subjected to structural elastic shortening and geometric non-linearities under extreme axial loads.

The third group, Geotechnical Resisting Capacity, circumvents the curse of dimensionality caused by numerous localized soil mechanics parameters by strategically utilizing Standard Penetration Test (SPT) N-values as comprehensive macro-level proxy variables. Specifically, this group incorporates the Average Shaft SPT N-value ($N_{sh}$) and the Pile Tip SPT N-value ($N_{t}$). To ensure major stratum contributions are properly prioritized and avoid simplistic arithmetic distortion, $N_{sh}$ is calculated as a thickness-weighted average based on the specific thicknesses of the intersected soil layers, effectively reflecting the relative density and overall shear strength along the pile shaft. While this macro-level proxy approach inherently smooths out localized layer-sequencing nuances, it successfully prevents dimensionality inflation when aggregating heterogeneous multi-source field databases. Meanwhile, $N_{t}$ quantifies the stiffness and ultimate bearing capacity of the founding stratum, serving as a critical boundary parameter to arrest plunging failure mechanisms when extreme axial loads are reached.

Finally, the Structural Material Stiffness group is represented by the Pile Elastic Modulus ($E_{s}$), completing the soil-structure interaction framework by defining the intrinsic axial rigidity of the structural member. In accordance with Hooke’s Law, incorporating $E_{s}$ provides the machine learning algorithm with a physical resisting boundary inversely proportional to the axial elastic shortening of the pile material.

All parameters are summarized and explained in Table 2.

**Table 2:** Statistical characteristics of variables used in the study.

No.	Symbol	Parameter	Unit	Physical meaning
**Input**				
1	P	Load applied at the pile top	kN	The main factor causing deformation and settlement
2	Ls	Length of pile embedded in soil	m	Determining the actual interaction range between the pile and the soil
3	Lf	Length of free pile section	m	Pile deformation without soil friction
4	Nsh	Average SPT value of the pile shaft	blow count	Characterizing the interaction between the pile and the surrounding soil layers
5	Nt	SPT value at the pile tip	blow count	Characterizes the soil behavior at the pile tip
6	O	Pile diameter	mm	Determines the contact area and cross-sectional area
7	Es	Pile elastic modulus	GPa	Stiffness of pile material
**Output**				
8	S	Pile top settlement	mm	Value to be predicted

In this study, the output variable (settlement S) is converted to a logarithmic scale (Log(S)) to minimize heterogeneity of variance, bring the data to a normal distribution, and support machine learning algorithms that converge more effectively in capturing the nonlinear behavior of piles under axial loading. To explain this, it can be seen that pile settlement can vary from very small at initial load levels to very large as the pile approaches failure. If S remains constant, large values will dominate the loss function, causing the model to focus on predicting large values while ignoring errors at small values. When using Log(S), the gap between settlement at low load levels and near-failure load levels is narrowed (from −1–2), allowing the model to handle different data ranges more equally. In addition, the relationship between load and pile head settlement of bored piles is not a straight line but a complex nonlinear curve. In mathematics, many physical phenomena follow exponential or power-based laws. Taking logarithms helps to linearize part of this relationship. This helps machine learning algorithms find the rules faster and more accurately. Finally, in practice, the settlement S must always be greater than 0. If the model is trained directly on the actual value of S, the model may sometimes predict negative values (physically absurd) at extremely low load levels.

Based on the statistical descriptors (Table 3) and exploratory data distributions (Fig 2), an exceptionally wide and heterogeneous data range is evident. Specifically, the expansive variation in the embedded pile length (L_s_, ranging from approximately 4.1 m to 76.0 m) and the applied axial load (P, spanning from 0 to 26250 kN) illustrates that the dataset encompasses a comprehensive spectrum of construction scales, from low-rise residential foundations to heavy-duty infrastructure piers. The remarkably high standard deviations (SD) observed across primary variables such as P, L_s_, and N_t_ frequently approach or exceed their respective mean values. This demonstrates the absence of data clustering around a specific pile typology, thereby confirming the high structural and geological diversity of the database. Concurrently, this extreme variance poses severe mathematical limitations for traditional linear regression models, rigorously justifying the necessity of advanced machine learning architectures to decipher this highly non-linear feature space. From a geomechanical perspective, the SPT N-value at the pile tip (N_t_) exhibits an SD of 122.67, directly mirroring its mean of 118.00. This indicates massive fluctuations in end-bearing conditions, transitioning from relatively compressible strata (N_t_ = 17.5) to virtually impenetrable hard rock (N_t_ = 400). Although the average shaft SPT index (N_sh_) presents a slightly more bounded variation, it still distinctly captures the non-linear and highly heterogeneous nature of the surrounding soil profiles. Regarding the predictive target, the logarithmic transformation of settlement, Log(S), effectively handles extreme boundary conditions. The inclusion of the minimum value of −5.00 (corresponding to an infinitesimal actual physical settlement of $S\approx 10^{-5}$ mm under zero-load or initial load states) is crucial, as it forces the algorithms to meticulously learn the initial linear-elastic contraction phase of the pile. Finally, the transformed statistical profileLog(S), characterized by a mean of 0.32 and an SD of 1.82, resolves the severe positive skewness typical of raw settlement magnitudes. This variance-stabilizing transformation ensures a more reasonable probability distribution, fundamentally mitigating systemic magnitude biases and accelerating convergence during the machine learning training phase.

**Table 3:** Statistical characteristics of variables used in the study.

	P (kN)	Ls (mm)	Lf (mm)	Nsh	Nt	D (mm)	Es (Gpa)	Log(S)
Min	0	4140.02	0	12.24	17.5	127	0.97	−5
Max	26250	76000	6644.64	273.82	400	2865.12	34.18	2.35
Mean	3480.50	14776.84	627.62	58	118	946.68	29.3	0.32
SD*	3417.78	16561.14	1565.23	57.28	122.67	495.07	5.1	1.82
Median	2668.93	9677.4	152.4	43.34	51.5	762	30.44	0.79

SD*: Standard Deviation.

**Fig 2:**
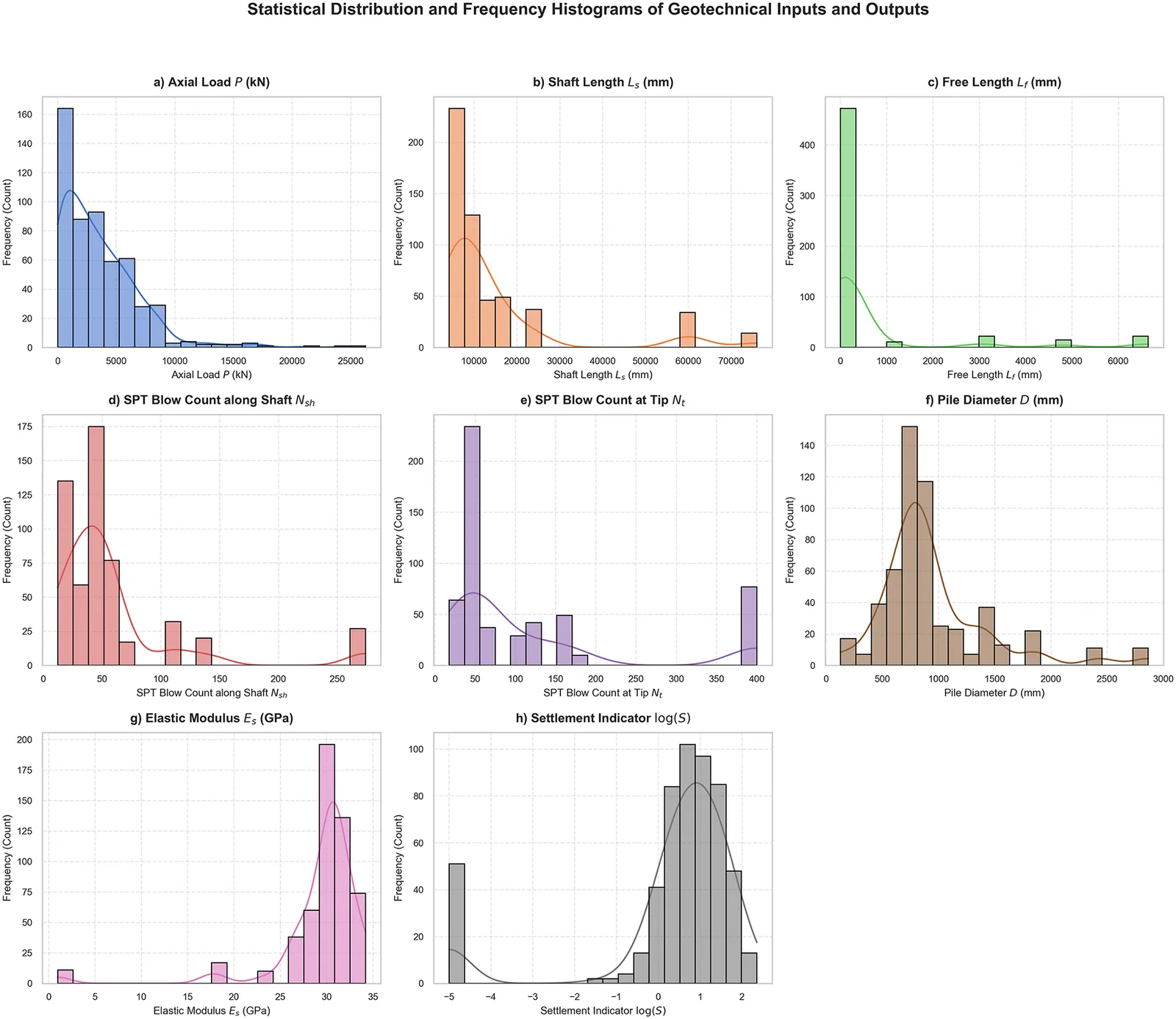
Exploratory data analysis and statistical distributions of the compiled geotechnical dataset: (a–g) Frequency histograms overlaid with continuous Kernel Density Estimation (KDE) curves for the independent input variables, and (h) Probability density profile of the dependent pile settlement target indicator log(S).

To ensure absolute objectivity and rigorously mitigate the risks of overfitting and data leakage, this study implements a stringent, multi-tiered data partitioning protocol. The compiled static load test (SLT) database is stochastically bifurcated into two primary partitions: a Training Pool (60%) and an Independent Testing Set (40%). As detailed in Table 4, the descriptive statistics confirm that this partitioning protocol successfully divides the 542 samples into a training pool (n = 325) and a strictly quarantined independent testing set (n = 217) while perfectly preserving population characteristics. Specifically, the identical extreme bounds (Min and Max) across both partitions – such as pile load (P up to 26,250 kN), side-friction length ($L_{s}$ up to 76,000 mm), and soil modulus ($E_{s}$) – verify that the independent test set encompasses the full geotechnical parameter space, eliminating extrapolation bias. Furthermore, the close alignment in central tendencies (Mean, Median) and dispersion (SD*) demonstrates that both subsets share an identical data distribution.

**Table 4:** Statistical characteristics of variables used in the study.

Dataset	Statistic	P (kN)	L_s_ (mm)	L_f_ (mm)	N_sh_	N_t_	D (mm)	E_s_ (Gpa)	Log(S)
Training set (Sample = 325)	**Min**	0.00	4140.02	0.00	12.24	17.50	127.00	0.97	−5.00
	**Max**	26250.00	76000.00	6644.64	273.82	400.00	2865.12	34.18	2.35
	**Mean**	3642.49	15790.37	634.58	55.62	117.90	957.68	29.30	0.39
	SD*	3760.39	17861.15	1569.97	54.17	123.87	505.84	5.16	1.77
	**Median**	2668.93	9677.40	152.40	39.42	51.50	812.80	30.44	0.80
Testing set (Sample = 217)	**Min**	0.00	4140.02	0.00	12.24	17.50	127.00	0.97	−5.00
	**Max**	16500.00	76000.00	6644.64	273.82	400.00	2865.12	34.18	2.21
	**Mean**	3237.89	13258.89	617.19	61.56	118.16	930.21	29.30	0.21
	SD*	2819.16	14302.32	1561.68	61.61	121.14	479.18	5.04	1.89
	**Median**	2668.93	9509.76	152.40	49.89	55.00	762.00	30.44	0.74

Crucially, the Testing Set is strictly quarantined throughout the entire model development and weight-tuning phases, functioning exclusively as an ultimate blind-test benchmark. Within the Training Pool, an internal nested validation structure is applied at an 80/20 ratio. Specifically, 80% of this pool is designated for training the intrinsic parameters of the heterogeneous base regressors (LR, RF, XGBoost, and MLP), while the remaining 20% acts as the Validation Set. This Validation Set plays a pivotal role by providing the objective loss metric – serving as the fitness function – that guides the two-stage hybrid optimization strategy (Random Search coupled with a Genetic Algorithm) to resolve the optimal continuous weight vector for the Weighted Ensemble architecture. Ultimately, enforcing this multi-stage isolation paradigm guarantees that the performance metrics derived from the Testing Set provide a mathematically unbiased reflection of the model’s true generalization capacity under highly variable, real-world geotechnical field conditions. Although the dataset size is compact (542 samples) – a common constraint in full-scale empirical geotechnical testing – the high structural diversity of the 51 distinct piles combined with our robust 40% independent blind test partition ensures that the model evaluates true generalization stability, effectively preventing small-sample overfitting without requiring computationally prohibitive cross-validation schemes like LOOCV.

To eliminate the adverse effects of heterogeneous measurement units and varying magnitude scales among input parameters, this study implements the Min-Max Scaling technique. This preprocessing step linearly transforms all continuous feature spaces into a uniform bound of [0, 1] prior to model training. This transformation significantly accelerates the mathematical convergence speed of the learning algorithms – particularly connectionist architectures like Multi-Layer Perceptrons (MLP) and gradient-boosted tree frameworks. Furthermore, it guarantees an equitable contribution weight of all independent variables to the final objective function, preventing features with large absolute ranges from exerting dominant bias. Crucially, to prevent data leakage and preserve evaluation integrity, the baseline scaling vectors – specifically the minimum (X_min_) and maximum (X_max_) values – are computed strictly from the Training Set. These frozen reference parameters are subsequently projected onto both the Validation Set and the independent Testing Set, ensuring that no statistical descriptors from the out-of-sample data contaminate the preprocessing pipeline. The standardized formula has the form:


$$\begin{array}{c} x^{\prime }=\frac{x-x_{min}}{x_{max}-x_{min}} \end{array}$$ (1)

In this formula, x is the original value of the variable; $x_{min}$ and $x_{max}$ are the minimum and maximum values of the variable in the dataset, respectively; $x^{\prime }$ is the variable’s value after normalization.

Following the rigorous data partitioning and scaling protocols, an exploratory feature dependency analysis was conducted using the Pearson correlation matrix (Fig 3). This mathematical diagnostic step is indispensable for verifying the statistical independence of the features (multicollinearity check) and quantifying the preliminary linear signals between the geotechnical predictors and the target variable, Log(S).

**Fig 3:**
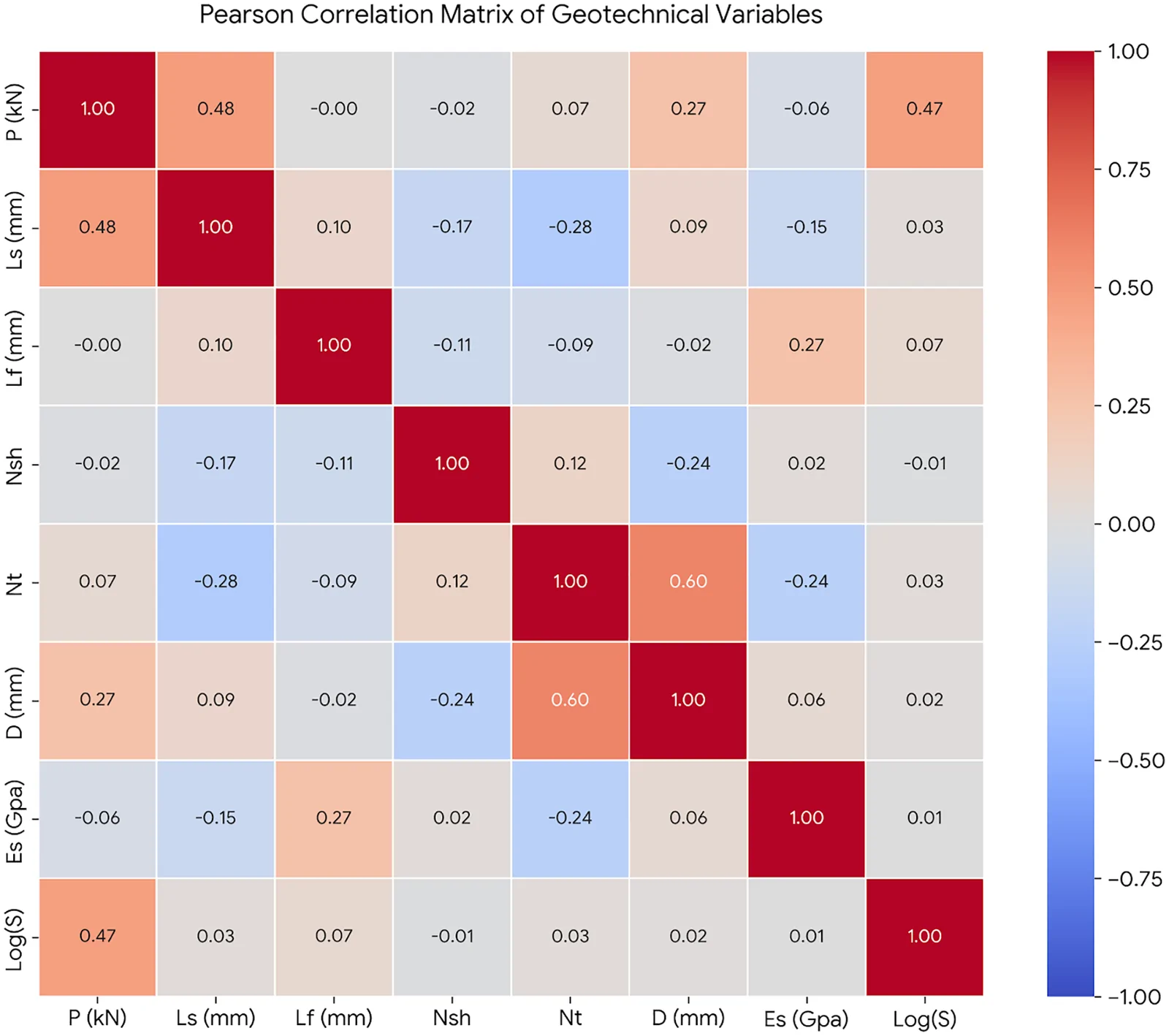
Exploratory feature dependency analysis: Pearson correlation heatmap illustrating linear interdependencies among structural dimensions, geotechnical parameters, and pile settlement.

As illustrated in Fig 3, the applied axial load (P) exhibits the most pronounced positive linear correlation with the settlement output (r = 0.47). From a geomechanical standpoint, this is fundamentally logical, as the external load acts as the primary driving mechanism for structural displacement. However, the moderate magnitude of this coefficient (0.47) distinctly indicates that the load alone cannot strictly dictate the settlement behavior; rather, the ultimate displacement is heavily governed by complex boundary interactions between the pile and the subsoil. Concurrently, the free-standing length ($L_{f}$) shows a marginal positive correlation (r = 0.07). While statistically weak in a linear sense, this physically translates to the elastic axial compression of the unconfined pile shaft acting as a free-standing column, a phenomenon that requires non-linear algorithms to fully capture its geometric P-Delta effects.

Conversely, the core geometric and geotechnical parameters ($N_{sh},N_{t},L_{s},D,E_{s}$) exhibit extremely weak linear correlations with the target variable (|r|<0.05). For instance, the average shaft resistance proxy ($N_{sh}$) registers a correlation of merely −0.01. Rather than implying a lack of physical importance, this statistical phenomenon perfectly encapsulates the highly non-linear and multidimensional nature of soil-structure interactions. For example, expanding the pile diameter (D) concurrently reduces the base contact stress while exponentially increasing the available surface area for skin friction mobilization. Such complex, polynomial interactions strictly evade the detection capabilities of the Pearson coefficient, which is mathematically constrained to identifying only straight-line relationships.

Furthermore, the matrix serves as a rigorous diagnostic tool for multicollinearity among the independent predictors. The highest inter-feature correlation is observed between the pile tip resistance ($N_{t}$) and the pile diameter (D) with r=0.60, followed by the correlation between applied load (P) and embedded length ($L_{s}$) at r=0.48. This reflects standard engineering heuristics: larger diameter piles are specifically selected to endure massive base resistance in hard strata, while extraordinarily heavy loads necessitate deeper pile embedment. Nonetheless, these correlation values remain comfortably below the critical collinearity threshold (r≥0.70), confirming that no severe multicollinearity afflicts the dataset. All seven selected features maintain an adequate degree of statistical independence, providing orthogonal information layers to the predictive models.

Although the compiled database combines international records from the FHWA repository with local field test samples from Vietnam – encompassing diverse geological formations ranging from dense coarse-grained profiles to coastal soft soils and alluvial clays – the input feature space is structurally harmonized using macro-level in-situ mechanical indicators (i.e., shaft and tip SPT N-values $N_{sh}$ and $N_{t}$, normalized pile geometries $L_{s}$, $L_{f}$, D, and material elastic modulus $E_{s}$). These parameters function as unified mechanical proxies, enabling the algorithms to evaluate pile-soil interaction based on fundamental stress-strain mechanics rather than purely regional descriptive classifications.

In summary, the correlation analysis unequivocally demonstrates that while the applied load initiates the settlement, the contributing geotechnical and geometric mechanisms are profoundly non-linear. The distinct absence of strong linear mapping serves as a profound theoretical justification for deploying advanced, non-linear machine learning algorithms – such as Random Forest, XGBoost, and the Multi-Layer Perceptron (MLP). These sophisticated architectures are mathematically indispensable to excavate and map the deeply hidden physical laws governing the pile settlement behavior, which traditional linear diagnostics completely fail to capture.

### 2.3. Base models development

#### 2.3.1. Linear regression (LR).

Linear Regression (LR) stands as a fundamental supervised machine learning algorithm extensively employed in engineering data analytics. The core objective of this architecture is to establish a strictly linear mapping between the dependent variable (target output, y) and a multi-dimensional set of independent variables (input features, x) by minimizing the residual variance through the Ordinary Least Squares (OLS) optimization technique [47]. In the context of predicting bored pile head settlement, the LR model operates on the underlying hypothesis that the total displacement S) can be mathematically approximated as a linear combination of the applied axial load, structural dimensions, and geotechnical soil properties, as governed by the following generalized equation:


$$\begin{array}{c} y=\beta_{\text{0}}+\beta_{\text{1}}x_{\text{1}}+\beta_{\text{2}}x_{\text{2}}+...+\beta_{n}x_{n}+\varepsilon \end{array}$$ (2)

Where:

y denotes the target predictive variable (pile head settlement); $x_{i}$ represents the independent geotechnical and structural input features.

$\beta_{\text{0}}$ is the intercept term (bias).

$\beta_{i}$ denotes the regression coefficients (weights), quantitatively defining the linear magnitude of influence exerted by each corresponding input parameter.

ε captures the stochastic residual error.

Despite its mathematical simplicity and high interpretability, the LR architecture is fundamentally constrained by its rigid assumption of linearity. This renders it inadequate for mapping the highly complex, non-linear deformation mechanisms inherent in real-world pile-soil interactions. Nevertheless, within the scope of this study, LR serves a strategic function as the baseline benchmark. Its inclusion establishes a rigorous comparative threshold, allowing for a quantitative evaluation of the accuracy enhancements and variance reductions achieved by the advanced non-linear ML algorithms and the proposed Weighted Ensemble framework.

#### 2.3.2. The multi-layer perceptron (MLP).

The Multi-Layer Perceptron (MLP) is a foundational feed-forward artificial neural network (ANN) architecture, widely recognized as a universal approximator capable of mapping extraordinarily complex, non-linear dependencies between input features and target outputs [48]. The topological structure of the MLPRegressor comprises at least three sequential computational stages: an input layer, one or multiple hidden layers, and a final continuous output layer. Applied to the bored pile settlement prediction problem, each neuron (node) within the hidden layers first executes an affine transformation on the incoming signals using a matrix of synaptic weights (w) and biases (b). This linearly transformed tensor is subsequently propagated through a strictly non-linear activation function – such as the Rectified Linear Unit (ReLU) or Hyperbolic Tangent (Tanh). This critical activation phase shatters the mathematical linearity of the algorithm, empowering the network to continuously extract and map the deep, non-linear geomechanical interactions governing the pile-soil system. The forward-propagation mechanism of the data through a specific computational node is generally expressed by the following mathematical formulation:


$$\begin{array}{c} z=\sigma \left(\sum_{i=1}^{n}w_{i}x_{i}+b\right) \end{array}$$ (3)

Where:

z denotes the output activation signal generated by the neuron.

f(·) represents the selected non-linear activation function.

$w_{i}$ encapsulates the synaptic weight vector connecting the adjacent computational nodes.

$x_{i}$ designates the input feature vector.

b stands for the internal bias term of the neuron.

The computational training phase of the MLP is driven by the Backpropagation algorithm coupled with gradient-based optimizers. This mathematical mechanism iteratively updates the synaptic weights and biases by computing the gradients to globally minimize a predefined loss function, conventionally the Mean Squared Error (MSE) [49]. The paramount advantage of the MLP architecture over traditional regression paradigms lies in its capacity for autonomous, deep feature extraction, completely bypassing the necessity for predefined parametric assumptions regarding the functional form of the data. However, to fully harness this non-linear predictive power, the network topology – specifically the depth (number of hidden layers) and width (nodal density per layer) – necessitates rigorous hyperparameter tuning. In the absence of such strict structural regularization, the MLP becomes highly susceptible to overfitting, which fundamentally compromises its out-of-sample generalization capabilities in practical foundation engineering applications.

#### 2.3.3. Random Forest (RF).

Random Forest (RF), formally introduced by Breiman [50], is a robust ensemble learning architecture rooted in the Bootstrap Aggregating (Bagging) paradigm. Operating as a decentralized meta-estimator, RF constructs a multitude of independent decision trees during the training phase. For continuous regression tasks such as pile settlement prediction, the ultimate predictive output is mathematically formulated as the arithmetic mean of the individual predictions yielded by the constituent trees. This stochastic aggregation mechanism fundamentally suppresses systemic variance and rigorously mitigates the risk of overfitting [42].

The architectural resilience of the RF model is governed by two core stochastic principles:

**Bootstrap Resampling:** Each constituent decision tree is independently trained on a stochastically drawn subset of the original training data (with replacement), ensuring high topological diversity among the trees.
**Feature Subspace Randomization:** At each nodal split, rather than evaluating the entire feature space, the algorithm evaluates only a random subset of input predictors to determine the optimal split. This decorrelates the individual trees, further enhancing the ensemble’s collective robustness.

Within the geotechnical domain, RF exhibits exceptional proficiency in navigating highly noisy geological datasets and mapping complex, high-dimensional soil-structure interactions. Furthermore, its intrinsic impurity-based feature importance metrics provide researchers with a quantitative diagnostic tool to evaluate the relative mechanical influence of varying stratigraphies and applied loads [51]. Nonetheless, a profound mathematical limitation of the tree-based RF architecture is its inherent inability to extrapolate; its predictive envelope is strictly bounded by the absolute minimum and maximum spatial limits of the training data. The generalized formulation of the RF regressor is expressed as:


$$\begin{array}{c} \hat{y}=\frac{\text{1}}{B}\sum_{b=1}^{B}T_{b}(x;\Theta_{b}) \end{array}$$ (4)

Where:

$\hat{y}$ denotes the aggregated ensemble predictive output (i.e., the estimated pile head settlement).

B represents the total number of independent decision trees cultivated within the forest architecture.

$T_{b}(\cdot )$ constitutes the continuous prediction generated by the $b^{th}$ individual tree estimator.

x designates the multi-dimensional geotechnical input feature vector.

$\Theta_{b}$ defines an independent and identically distributed (i.i.d.) stochastic parameter vector governing the structural topology of the $b^{th}$ tree. This vector explicitly dictates both the bootstrap data resampling mechanism and the randomized feature subspace selection at the nodal splits.

#### 2.3.4. XGBoost (XGB).

XGBoost, formally introduced by Chen and Guestrin [52], is a highly scalable and mathematically optimized implementation of the Gradient Boosting Decision Tree (GBDT) framework. In stark contrast to the parallel stochastic aggregation (Bagging) paradigm of Random Forest, XGBoost operates on a sequential, additive boosting architecture. In this strict algorithmic regime, each successive decision tree is purposefully cultivated not to predict the target independently, but to iteratively model and neutralize the pseudo-residuals (gradients of the loss function) left by the preceding ensemble. This process essentially executes a gradient descent optimization directly within the function space, aggressively driving the system toward the global minimum of a regularized objective function [53]. Mathematically, the additive predictive output of the XGBoost architecture at the $t^{th}$ boosting iteration is formulated by integrating the cumulative prediction from the prior step (t−1) with a newly parameterized tree function ($f_{t}$):


$$\begin{array}{c} {\hat{y}}_{i}^{\left(t\right)}={\hat{y}}_{i}^{\left(t-1\right)}+{\eta f}_{t}\left(x_{i}\right) \end{array}$$ (5)

Where:

${\hat{y}}_{i}^{\left(t\right)}$ denotes the cumulative predictive output for the $i^{th}$ data instance at the $t^{th}$ boosting iteration.

${\hat{y}}_{i}^{\left(t-1\right)}$ represents the inherited cumulative prediction from the preceding t−1 iterations.

η signifies the learning rate, or shrinkage factor. This parameter restricts the step-size contribution of each newly integrated tree, thereby decelerating the learning process to rigorously prevent overfitting; it is conventionally bounded within the interval [0,1].

$f_{t}\left(x_{i}\right)$ is the spatial predictive function of the independent decision tree newly parameterized and added to the ensemble at step t.

The core architectural superiority that distinguishes XGBoost from traditional GBDT frameworks lies in the direct integration of an explicit Regularization mechanism into its Objective Function. This mechanism establishes a rigorous mathematical boundary, proactively constraining the structural complexity of the trees to fundamentally eradicate overfitting:


$$\begin{array}{c} Obj\left(\phi \right)=\sum_{i}L\left(y_{i},{\hat{y}}_{i}\right)+\sum_{k}\Omega \left(f_{k}\right) \end{array}$$ (6)

Where:

Obj is the overarching objective function to be globally minimized.

L represents a strictly differentiable loss function, quantitatively measuring the deviation between the model’s predictions and the actual ground-truth settlement.

Ω constitutes the regularization penalty term. It explicitly penalizes the model for cultivating trees with an excessive number of terminal leaves or extreme leaf weights, thereby forcing the architecture to maintain a minimalist topology and dramatically enhancing its out-of-sample generalization capacity.

From a systems engineering perspective, XGBoost deploys a block-level parallel computing mechanism coupled with a histogram-based split-finding algorithm. This architectural breakthrough yields extraordinarily fast convergence rates and superior interpolation efficacy on massive, complex geotechnical datasets [54]. Specifically for the bored pile settlement problem, XGBoost exhibits remarkable resilience against highly skewed input distributions and demonstrates a formidable capacity to map high-order, non-linear interactions among the pile structure, multi-layered subsoils, and applied axial loads. Consequently, embedding XGBoost into the proposed Weighted Ensemble architecture serves a profound strategic purpose: it operates as an algorithmic “residual hunter,” specialized in identifying and compensating for residual discrepancies within anomalous data zones – spatial domains where linear algorithms and standalone models chronically fail.

### 2.4. Weighted ensemble architecture

Standalone base models have been widely deployed in geotechnical studies analyzing pile bearing capacity and settlement behavior. However, from a mathematical standpoint, the out-of-sample performance of any single architecture is strictly governed by the fundamental bias-variance tradeoff. Specifically, the Linear Regression (LR) architecture establishes a baseline characterized by low variance and high bias. This intrinsic property renders LR virtually immune to overfitting, yet it is fundamentally inadequate for approximating complex, non-linear data spaces. Conversely, Random Forest (RF) and the Multi-Layer Perceptron (MLP) exhibit low bias but high variance; they possess exceptional capabilities in mapping deep, non-linear geomechanical interactions, yet are acutely sensitive to data noise, leading to severe accuracy degradation on out-of-sample domains. Meanwhile, XGBoost, despite being equipped with a robust optimization mechanism, still harbors the risk of systemic dispersion if its topology is not perfectly regularized. Stemming from these algorithmic diagnostics, the proposed Weighted Ensemble framework emerges as a holistic, globally optimized paradigm. This architecture transcends naive arithmetic averaging to function as an intricate mechanism of cross-residual compensation. By allocating a precisely tuned continuous weight vector ([w]), the ensemble framework maximizes the non-linear predictive power of the complex architectures (RF, MLP, XGBoost) while strategically leveraging the structural stability of the linear model (LR) as a mathematical damping anchor. The result is a hybrid system that achieves an absolute equilibrium: it possesses sufficient mathematical complexity to exhaustively map the physical laws governing pile-soil interactions, whilst maintaining strict structural parsimony to fundamentally eradicate the risk of overfitting. To preserve absolute statistical objectivity and completely isolate the efficacy of the optimization algorithm – thereby preventing any risk of hyperparameter-induced data leakage – all base regressors in this study were configured using their respective optimized hyperparameters (derived from the Randomized Search phase detailed in Section 3.1) and held frozen throughout the ensemble weight optimization process. The computational pipeline is executed as follows: after the independent training phase, each base estimator generates a primary predictive vector. In the ensuing phase, these independent vectors are extracted and aggregated to form the input feature matrix for the ensemble meta-estimator. Quantitatively, the final predictive output of the Weighted Ensemble architecture is governed by the following mathematical formulation:


$$\begin{array}{c} {\text{y}}_{\text{ens}emble}=\sum_{i=1}^{n}{\text{w}}_{i}.y_{i} \end{array}$$ (7)

Where:

$y_{ensemble}$ denotes the aggregated predictive output of the ensemble architecture.

$w_{i}$ represents the continuous weight assigned to the $i^{th}$ base estimator.

$y_{i}$ constitutes the individual predictive output generated by the $i^{th}$ base estimator.

The assigned weights are strictly bounded within the interval $w_{i}\in \left[0,1\right]$ and must mathematically satisfy the equality constraint $\sum w_{i}=1$. This normalization ensures that the spatial contribution of the constituent models remains dimensionally consistent, establishing a probability-like distribution of algorithmic importance.

To resolve the optimal configuration of the continuous weight vector (𝐰), a robust two-stage hybrid optimization strategy is implemented, coupling Random Search (RS) with a Genetic Algorithm (GA)(the full optimization and modeling source code is provided in S2 File). Both metaheuristic frameworks inherently facilitate global optimization and completely bypass the mathematical prerequisite of gradient continuity within the objective function. While RS offers superior computational velocity by aggressively exploring the feasible multi-dimensional weight space via stochastic sampling, the GA excels in deep exploitation. It systematically inherits elite weight combinations from preceding generations, applying evolutionary operators (crossover and mutation) to iteratively refine and breed mathematically superior topological solutions for subsequent generations. Consequently, this study architecturally fuses RS and GA to synergize their respective exploratory (global search) and exploitative (local fine-tuning) strengths. Mechanistically, the RS algorithm is initially deployed as a global scout to rapidly navigate the vast mathematical weight space.

Following the global exploration phase, rather than initializing the GA population with purely stochastic values, a constrained spatial refinement step is executed. Specifically, the algorithm extracts the top 100 elite weight configurations exhibiting the lowest Mean Absolute Error (MAE) from the RS phase. From this archive of elite solutions, the absolute marginal boundaries (minimum and maximum values) for each individual weight $w_{i}$ are explicitly defined. The initial population of the GA is subsequently formulated by uniformly distributing individuals strictly within these established boundaries. This boundary-restriction strategy effectively narrows the search scope into an ‘optimal basin,’ empowering the GA to completely bypass suboptimal weight combinations. Consequently, the algorithmic computational power is exclusively concentrated on localized fine-tuning to resolve the final optimal weight vector. Ultimately, this sequential hybridization rigorously immunizes the system against premature convergence into local minima. Concurrently, it drastically accelerates the mathematical convergence trajectory of the GA compared to a standalone evolutionary approach. This synergistic mechanism significantly mitigates the overall computational latency – a critical structural advantage when simultaneously training a complex matrix of machine learning architectures.

**Algorithm: Hybrid RS-GA Optimization for Weighted Ensemble**

**Input:**

Geotechnical dataset 𝒟={X,y}

Set of trained base estimators $ℳ=\{M_{\text{1}},M_{\text{2}},\ldots ,M_{K}\}$

Hyperparameters for Random Search ($N_{\text{RS}}$) and Genetic Algorithm ($N_{\text{gen}}$)

**Output:**

Optimal weight vector ${𝐰}^{*}=[w_{\text{1}},w_{\text{2}},\ldots ,w_{K}{]}^{T}$

Final evaluation metrics on the independent test set

**Phase 1: Data Preparation & Base Initialization**

1: Split dataset 𝒟 into training subset ${𝒟}_{\text{train}}$ and testing subset ${𝒟}_{\text{test}}$

2: Normalize feature space $X_{\text{train}}$ to $X_{\text{train}}^{\text{norm}}$ using Min-Max Scaler

3: **for each** model $M_{k}\in ℳ$ **do**

4:  Train $M_{k}$ exclusively on ${𝒟}_{\text{train}}$

5:  Generate validation predictive vector: ${\hat{𝐲}}_{k}=M_{k}\left(X_{\text{train}}^{\text{norm}}\right)$

6: **end for**

**Phase 2: Random Search (RS) Global Exploration**

7: Initialize minimum loss ${ℒ}_{min}\leftarrow \infty$, elite seed vector ${𝐰}_{\text{elite}}\leftarrow \emptyset$, and archive 𝒜←∅

8: **for** i = 1 to $N_{\text{RS}}$ **do**

9:  Sample random candidate vector ${𝐰}^{\left(i\right)}\in {\mathbb{R}}^{K}$ from a uniform distribution

10:  Enforce mathematical constraints: $\sum_{k=1}^{K}w_{k}^{\left(i\right)}=1$ and $0\leq w_{k}^{\left(i\right)}\leq 1$

11:  Compute ensemble output: ${\hat{𝐲}}_{\text{ens}}=\sum_{k=1}^{K}w_{k}^{\left(i\right)}{\hat{𝐲}}_{k}$

12:  Compute loss function (MAE): ${ℒ}^{\left(i\right)}=\frac{\text{1}}{N}\sum |y-{\hat{y}}_{\text{ens}}|$

13:  Add $\left({𝐰}^{\left(i\right)},{ℒ}^{\left(i\right)}\right)$ to archive 𝒜

14:  **if** ${ℒ}^{\left(i\right)}<{ℒ}_{min}$ **then**

15:   ${ℒ}_{min}\leftarrow {ℒ}^{\left(i\right)}$

16:   ${𝐰}_{\text{elite}}\leftarrow {𝐰}^{\left(i\right)}$

17:  **end if**

18: **end for**

19: Extract top 100 elite vectors from 𝒜 with the lowest MAE to determine bounds ${𝐰}_{\text{bounds}}$

**Phase 3: Genetic Algorithm (GA) Refinement**

20: Initialize population ${𝒫}_{\text{0}}$ by sampling within ${𝐰}_{\text{bounds}}$ and incorporating ${𝐰}_{\text{elite}}$

21: **for** gen = 1 to $N_{\text{gen}}$ **do**

22:  **for each** individual $𝐰\in {𝒫}_{\text{gen}-1}$ **do**

23:   Compute fitness function: $\text{Fitness}\left(𝐰\right)=\left(\frac{\text{1}}{1+ℒ\left(𝐰\right)}\right)$

24:  **end for**

25:  Select parent chromosomes from ${𝒫}_{\text{gen}-1}$ based on fitness proportionate probability

26:  Apply simulated binary crossover and polynomial mutation operators within ${𝐰}_{\text{bounds}}$

27:  Enforce normalization constraint: $\sum w_{k}=1$

28:  Update population to form ${𝒫}_{\text{gen}}$

29: **end for**

30: Extract the globally fittest individual ${𝒫}_{N_{\text{gen}}}$ as the optimal weight vector ${𝐰}^{*}$

### 2.5. Performance evaluation

To rigorously evaluate the predictive fidelity of the proposed machine learning architectures, a comprehensive suite of statistical performance metrics is utilized. Relying on a single mathematical indicator is fundamentally insufficient; therefore, these metrics must be deployed concurrently to provide a holistic assessment of the model’s accuracy, stability, and generalization capacity.

The Mean Absolute Error (MAE) computes the arithmetic average of the absolute residuals, applying a strictly linear penalty to predictive errors. This metric provides a robust representation of the actual average deviation without being disproportionately skewed by sporadic data anomalies. Conversely, the Root Mean Squared Error (RMSE) squares the residuals prior to averaging, thereby imposing a severe mathematical penalty on significant predictive outliers. Minimizing the RMSE is exceptionally critical in geotechnical applications, where large localized prediction failures can lead to catastrophic foundation design errors.

Furthermore, the Coefficient of Determination ($R^{\text{2}}$) quantifies the proportion of the target variable’s variance that is successfully captured by the algorithmic model, effectively verifying its capability to map the global non-linear data trends. Crucially, from a strict engineering perspective, a model might yield an ostensibly high $R^{\text{2}}$ value while still generating absolute physical errors (MAE, RMSE) that exceed safe structural tolerances. Thus, an architecturally sound model must simultaneously maximize the $R^{\text{2}}$ coefficient whilst strictly minimizing both MAE and RMSE.

However, while MAE and RMSE provide indispensable engineering insights into the absolute physical magnitude of prediction residuals, their mathematical formulations remain intrinsically scale-dependent. Within heterogeneous geotechnical datasets, structural pile geometries (e.g., embedded lengths, diameters) and extreme loading scenarios vary extensively, causing the observed physical settlements (S) to span vastly different numerical magnitudes. Consequently, evaluating the model’s generalized performance across diverse data subsets using standalone absolute metrics can introduce severe interpretation biases. To circumvent this systemic scale limitation and establish an objective, scale-agnostic validation benchmark, the Normalized Root Mean Squared Error (NRMSE) is introduced as a core comparative diagnostic metric.

The mathematical formulations governing these evaluation criteria are defined as follows:

The Coefficient of Determination ($R^{\text{2}}$):


$$\begin{array}{c} R^{\text{2}}=1-\frac{{SS}_{res}}{{SS}_{tot}}=1-\frac{\sum_{i=1}^{n}(y_{i}-{\hat{y}}_{i}{)}^{\text{2}}}{(y_{i}-\overset{―}{y}{)}^{\text{2}}} \end{array}$$ (8)

Where:

${SS}_{res}$ denotes the Residual Sum of Squares, mathematically quantifying the unexplained variance of the model (i.e., the cumulative squared deviations between the algorithmic predictions and the actual observations).

${SS}_{tot}$ represents the Total Sum of Squares, defining the intrinsic baseline variance of the dataset relative to its arithmetic mean.

$y_{i}$ signifies the actual ground-truth settlement recorded in the dataset.

${\hat{y}}_{i}$ is the corresponding settlement output predicted by the implemented model.

$\overset{―}{y}$ denotes the arithmetic mean of all observed ground-truth values.

n represents the total number of data instances evaluated within the subset.

Diagnostic Characteristics of the Coefficient of Determination ($R^{\text{2}}$):

Theoretically, the $R^{\text{2}}$ metric is bounded within the interval (−∞,1], carrying profound statistical implications:

$R^{\text{2}}$ = 1.0 indicates a mathematically perfect predictive mapping, signifying that the model completely captures 100% of the variance within the target data. $R^{\text{2}}$ = 0.0 implies that the algorithmic predictive capability is strictly equivalent to a naive baseline estimator (i.e., a model that statically predicts the arithmetic mean $\overset{―}{y}$ for all incoming inputs, regardless of feature variations). $R^{\text{2}}$ < 0.0 manifests specifically in non-linear or mis-specified architectures where the predictive performance is demonstrably inferior to the naive mean baseline. This indicates a severe structural failure in mapping the physical data trends or an extreme case of algorithmic extrapolation error.

- The Mean Absolute Error (MAE):

$$\begin{array}{c} MAE=\frac{\text{1}}{n}\sum_{i=1}^{n}\left|y_{i}-{\hat{y}}_{i}\right| \end{array}$$ (9)

Diagnostic Characteristics: The MAE mathematically quantifies the average magnitude of the predictive residuals across the dataset, strictly disregarding their directional signs (i.e., whether the errors are positive or negative). Consequently, a minimized MAE value explicitly indicates a lower average absolute deviation, which directly translates to superior predictive fidelity and overall algorithmic efficacy.

- The Root Mean Squared Error (RMSE):

$$\begin{array}{c} RMSE=\sqrt{\frac{\text{1}}{n}\sum_{i=1}^{n}(y_{i}-{\hat{y}}_{i})} \end{array}$$ (10)

Diagnostic Characteristics: Driven by its inherent squaring mechanism, the RMSE imposes a disproportionate mathematical penalty on large-magnitude residuals. Consequently, a substantial divergence between the RMSE and the MAE serves as a critical algorithmic diagnostic: it unequivocally indicates that the model’s predictive envelope is severely compromised by extreme, localized outliers (i.e., catastrophic predictive failures).

- The Normalized Root Mean Squared Error (NRMSE):

$$\begin{array}{c} NRMSE=\frac{RMSE}{y_{max}-y_{min}}\mathrm{.100}\% \end{array}$$ (11)

Where:

$y_{max}$ and $y_{min}$ denote the absolute maximum and minimum displacement values recorded within that corresponding dataset partition.

Diagnostic Characteristics: By mathematically normalizing the absolute RMSE against the statistical dispersion (typically the range or mean) of the ground-truth observations, the NRMSE effectively neutralizes inherent scale-dependencies. This transformation yields a strictly dimensionless relative metric. Consequently, a minimized NRMSE explicitly demonstrates that the algorithmic architecture maintains a consistent proportional accuracy regardless of the data’s numerical magnitude. This renders the NRMSE an indispensable benchmark for impartially evaluating and cross-validating the model’s generalization capabilities across highly heterogeneous geotechnical scenarios (e.g., varying structural pile geometries and extreme loading conditions)

## 3. Results and discussions

### 3.1. Hyperparameter space optimization for base estimators via randomized search

The predictive capacity and generalization robustness of any machine learning architecture are strictly governed by the fine-tuning of its internal hyperparameters. To determine the optimal topological configuration for each base estimator, this study implements a Randomized Search (RS) strategy [55], rigorously coupled with a 10-fold Cross-Validation [42] mechanism on the training dataset. In stark contrast to Grid Search (GS), which relies on an exhaustive deterministic search mechanism and is fundamentally susceptible to combinatorial explosion and severe computational resource depletion, the RS algorithm executes stochastic sampling of parameter vectors from a predefined probability distribution space. This stochastic sampling mechanism has been mathematically proven to deliver superior efficacy in navigating high-dimensional feature spaces. It accelerates the system’s convergence trajectory toward global optima while drastically minimizing computational overhead [55]. To establish an optimal equilibrium between expansive spatial exploration and localized exploitation, the computational budget for the RS algorithm in this study was strictly capped at 300 iterations. The search space for hyperparameters for each model is shown in **Table 5**.

**Table 5:** Hyperparameter search space for baseline models and optimal results.

Model	Hyperparameter	Symbol	Range	Optimal value	Optimum R2 score
**Random Forest (RF)**	Number of trees	n_estimators	[50, 300]	160	0.651
	Maximum depth of the tree	max_depth	[2, 20]	14	
	Minimum number of samples to split	min_samples_split	[2, 10]	7	
**XGBoost (XGB)**	Number of iterations	n_estimators	[50, 300]	232	0,65
	Learning rate	learning_rate	[0.01, 0.3]	0.087	
	Maximum depth of the tree	max_depth	[2, 10]	3	
**MLP Regressor**	Hidden layer architecture	hidden_layer_sizes	[1, 2] hidden layers; [10, 100] neurons per layer	[20]	0.696
	Regularization coefficient (L1)	alpha	[10e-5, 10]	0.02	
	Initial learning rate	learning_rate_init	[0.001, 0.1]	0.083	
	Activation function	activation	ReLU (1), Tanh (2), Logistic (3)	ReLU	
	Training algorithm	solver	ADAM (1), LBFGS (2), SGD (3)	LBFGS	

The comprehensive results of the hyperparameter optimization phase are summarized in **Table 5**. Through the deployment of the Randomized Search (RS) algorithm, the specific convergence trajectories and optimal parameter basins for each base architecture were systematically identified and visualized via parallel coordinates plots, detailed as follows:

- **Random Forest (RF):** The RS algorithm successfully converged to the global optimum at the 12th iteration, yielding an $R^{\text{2}}$ score of 0.651. Furthermore, as explicitly illustrated by the parallel coordinates plot in Fig 4, the broader stochastic exploration revealed that the RF architecture maintains robust predictive stability when n_estimators is bounded within [100, 200], min_samples_split is restricted to [6,9], and max_depth is strictly constrained within [12,18].
- **XGBoost (XGB):** The extreme Gradient Boosting model achieved its peak topological configuration at iteration 71, recording an $R^{\text{2}}$ of 0.650. The empirical data, mapped in Fig 5, indicates a highly logical operational basin for XGBoost when n_estimators is configured within [120, 300], the learning_rate is regulated between [0.15, 0.25], and the max_depth is kept structurally shallow between [2,4].
- **Multi-Layer Perceptron (MLP):** Characterized by a significantly more intricate hyperparameter space, the MLP reached its absolute optimum at iteration 84, delivering a superior $R^{\text{2}}$ of 0.696. Notably, the variance in the $R^{\text{2}}$ spectrum for the MLP across different spatial configurations was substantially wider compared to the tree-based models, drastically fluctuating from −0.028 to 0.696. This profound volatility unequivocally demonstrates the MLP’s acute hypersensitivity to topological tuning; arbitrary or suboptimal parameter selection intrinsically leads to catastrophic predictive degradation. Ultimately, the RS trajectory, as geometrically depicted in the parallel coordinates plot (Fig 6), elucidates that the MLP maximizes its pile settlement prediction capacity when deploying the LBFGS weight optimizer and the ReLU activation function. The network performs exceptionally well when the hidden neuron count per layer is restricted within the [10,40] range, with the absolute peak performance empirically isolated using a minimalist topology of a single hidden layer equipped with precisely 20 computational nodes.

**Fig 4:**
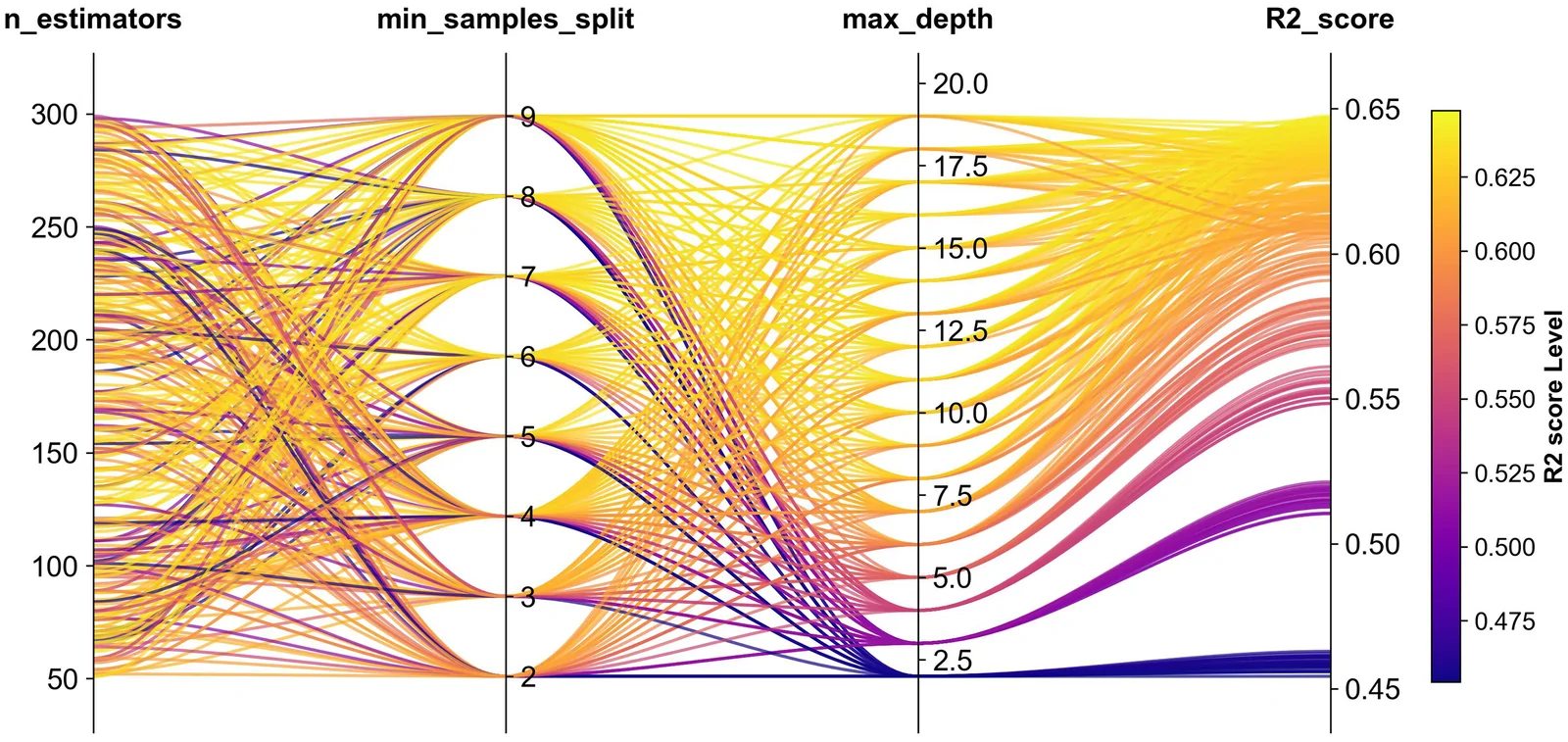
Parallel coordinates visualization of the hyperparameter search space and ${𝐑}^{2}$ performance for the RF model via Randomized Search.

**Fig 5:**
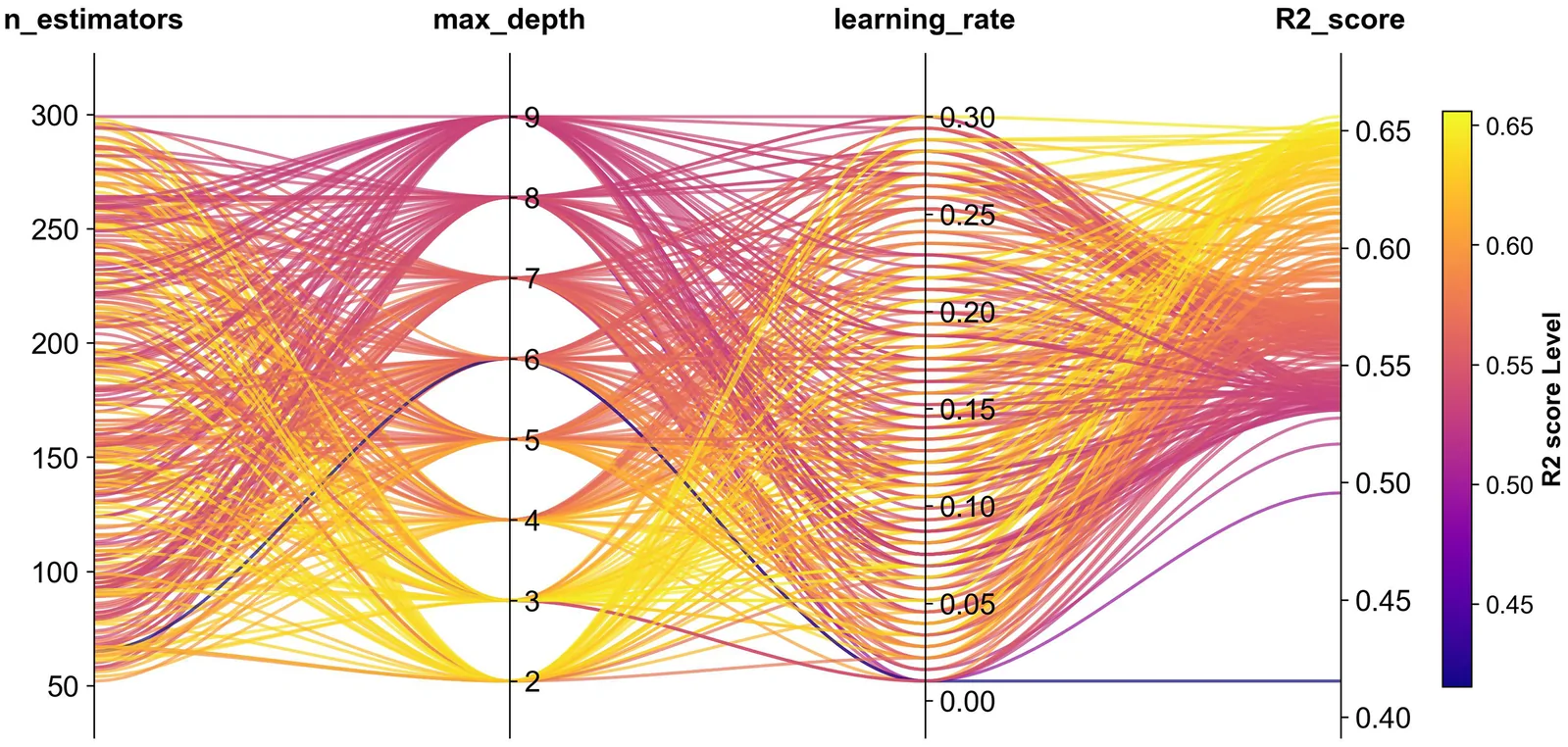
Parallel coordinates visualization of the hyperparameter search space and performance for the *XGB* model via Randomized Search.

**Fig 6:**
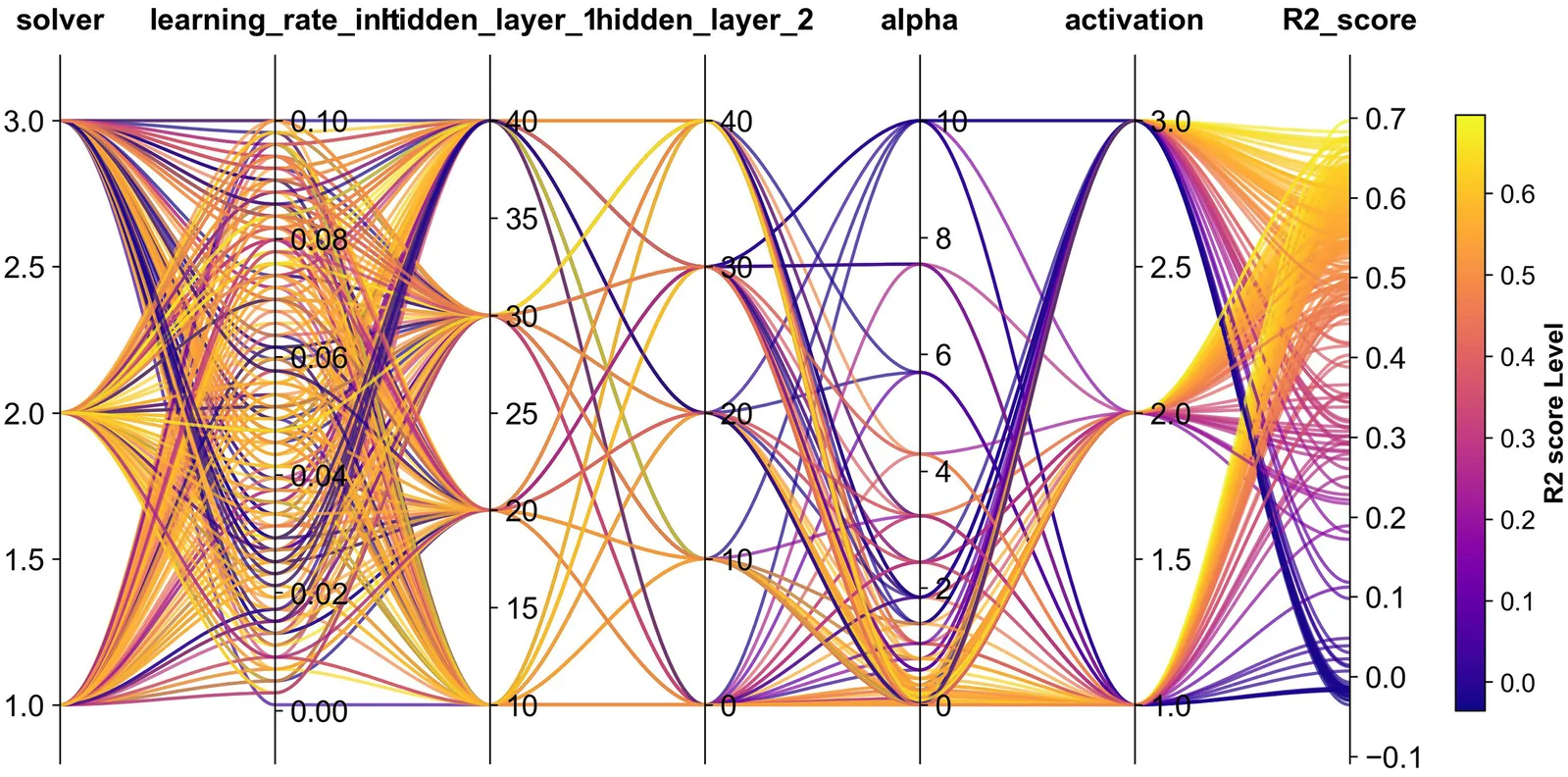
Parallel coordinates visualization of the hyperparameter search space and ${𝐑}^{2}$ performance for the MLP architecture via Randomized Search.

### 3.2. Performance evaluation and comparative analysis of baseline models

Following the rigorous hyperparameter optimization executed exclusively on the training dataset, the predictive performance of the four optimized baseline machine learning models - Linear Regression (LR), Random Forest (RF), eXtreme Gradient Boosting (XGBoost), and Multi-Layer Perceptron (MLP) – was systematically evaluated. To ensure strict methodological integrity and prevent any potential data leakage, the validation and testing datasets were kept entirely sequestered (unseen) during both the algorithmic training and hyperparameters tuning phases. Consequently, the predictive metrics derived from these two hold-out subsets provide a strictly objective and unbiased representation of the base models’ true out-of-sample generalization capabilities.

Fig 7 graphically illustrates the regression scatter matrices, directly mapping the actual experimental observations against the algorithmic predictions across all datasets. These visualizations are comprehensively accompanied by the core statistical evaluation metrics: the Coefficient of Determination ($R^{\text{2}}$), Mean Absolute Error (MAE), Root Mean Squared Error (RMSE), and Normalized Root Mean Squared Error (NRMSE). The results are also summarized in **Table 6**.

**Table 6:** Summary of performance of comprehensive base models.

Dataset	Model	MAE	RMSE	R2	NRMSE
Training set	Linear Regression	1.0247	1.5281	0.2953	20.78%
	Random Forest	0.0778	0.1104	0.9963	1.50%
	XGBoost	0.1103	0.1492	0.9933	2.03%
	MLP Regressor	0.2144	0.3211	0.9689	4.37%
Validation set	Linear Regression	0.859	1.2359	0.3647	16.93%
	Random Forest	0.2259	0.34	0.9519	4.66%
	XGBoost	0.2092	0.285	0.9662	3.91%
	MLP Regressor	0.2689	0.3613	0.9457	4.95%
Testing set	Linear Regression	1.0349	1.6314	0.2268	22.63%
	Random Forest	0.2036	0.4231	0.948	5.87%
	XGBoost	0.2316	0.4039	0.9526	5.60%
	MLP Regressor	0.2969	0.5075	0.9252	7.04%

**Fig 7:**
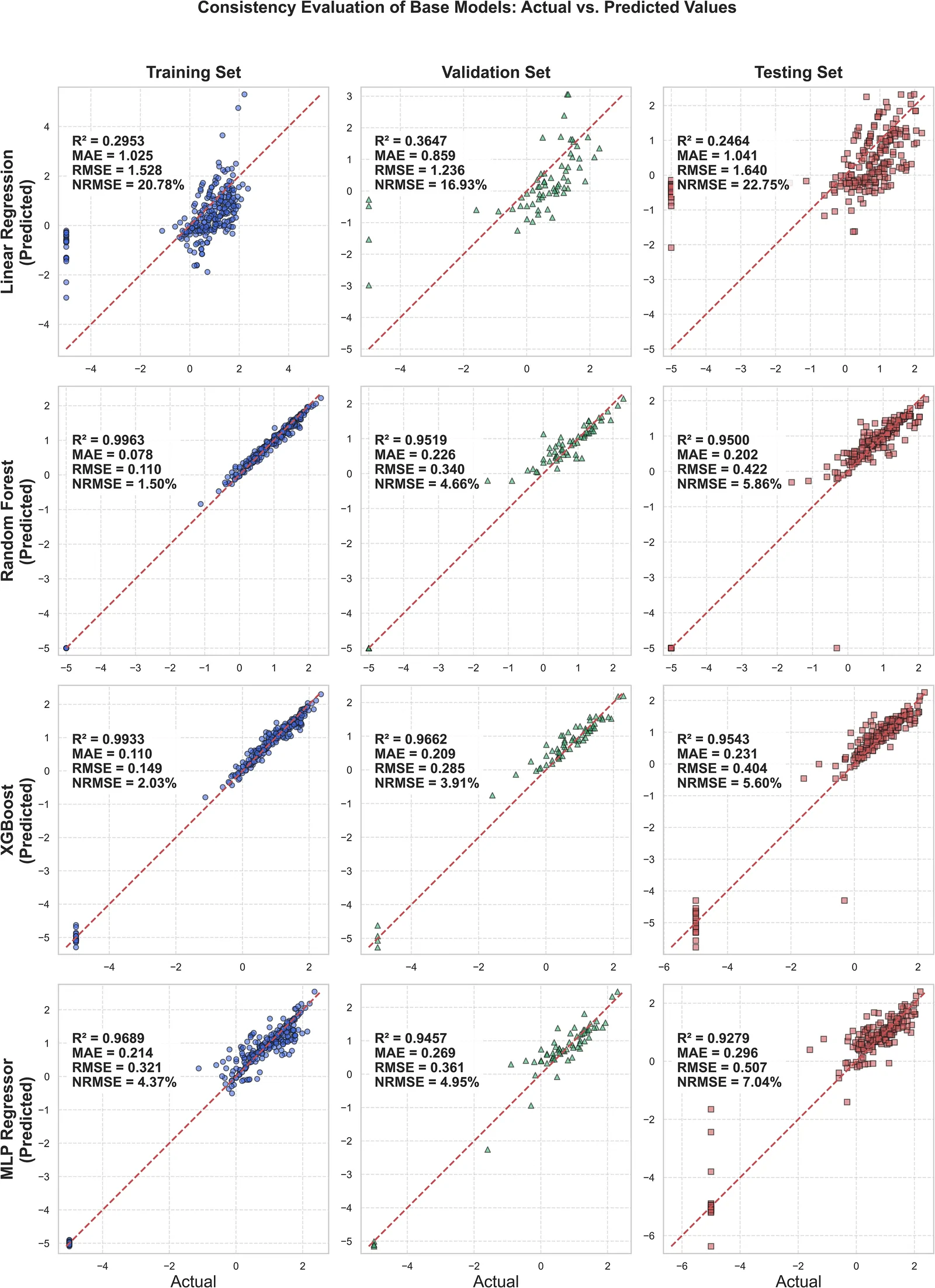
Regression scatter matrices illustrating the predictive consistency of the four baseline models (LR, RF, XGBoost, MLP) across the training, validation, and testing datasets.

Initial observations explicitly demonstrate that the **Linear Regression (LR)** model suffers from profound structural and data-level mismatches. Exhibiting a severe lack of predictive capacity, the LR algorithm yielded an $R^{\text{2}}$ of merely 0.2268 and an NRMSE of 22.63% on the independent testing set. The highly dispersed scatter plots across all three data partitions unequivocally indicate that a rigid linear paradigm is fundamentally incapable of capturing the complex, non-linear mappings inherent in pile-soil interaction mechanics. This pronounced residual variance confirms that dynamic load-settlement behaviors cannot be simplified into linear boundary conditions, intrinsically leading to severe underfitting.

In stark contrast to the linear approach, the ensemble tree-based **Random Forest (RF)** architecture demonstrated exceptional predictive accuracy and robust variance control. The RF model achieved an $R^{\text{2}}$ of 0.9963 during the training phase, successfully translating into a highly stable $R^{\text{2}}$ of 0.9480 and an NRMSE of 5.87% on the unseen testing set. The tight clustering of predicted data points along the ideal 1:1 reference line implies that its inherent bagging (bootstrap aggregating) mechanism effectively mitigates overfitting, thereby ensuring strong generalization capabilities when introduced to new geotechnical conditions.

Among the evaluated architectures, **XGBoost** emerged as the premier individual baseline model within this comparative framework. It established the lowest testing error metrics (MAE = 0.232, RMSE = 0.404, and NRMSE = 5.60%), coupled with a superior testing $R^{\text{2}}$ of 0.9526. The algorithm’s iterative gradient boosting mechanism sequentially minimizes residual errors across successive trees, rendering the model highly adept at mapping localized geotechnical variations and complex non-linear structural degradation curves under peak loading states.

Furthermore, the connectionist **Multi-Layer Perceptron (MLP)** architecture demonstrated a strong capacity for continuous non-linear mapping, yielding a satisfactory testing $R^{\text{2}}$ of 0.9252 and an NRMSE of 7.04%. Although this neural network successfully resolved the macro-level non-linear trends of the dataset, a noticeable spatial dispersion occurred at lower settlement magnitude zones (i.e., the lower-left quadrant of the testing matrix). This elevated residual variance suggests that while the MLP excels at global function approximation, it remains relatively sensitive to localized noise and data sparsity when constrained by the small-scale sample distributions typical of geotechnical datasets.

While the advanced non-linear baseline models – specifically XGBoost and RF – demonstrated commendable predictive fidelity with $R^{\text{2}}$ scores exceeding 0.94, relying strictly on a solitary algorithmic architecture introduces inherent epistemic uncertainties. In geotechnical engineering, pile-soil interactions are governed by highly heterogeneous soil stratigraphy, complex stress-strain histories, and non-linear consolidation behaviors. Consequently, a single model, regardless of rigorous hyperparameter optimization, retains an inescapable structural bias dictated by its intrinsic mathematical formulation. For instance, tree-based models (RF and XGBoost) partition the feature space into discrete orthogonal hyper-rectangles, potentially inducing localized step-wise discontinuities in the predicted load-settlement curves. Conversely, connectionist models (MLP) approximate continuous functions globally but are notoriously susceptible to localized noise and varying data densities. Therefore, an uncompensated reliance on any individual baseline model poses a systemic risk of localized predictive failures, particularly under extreme loading states or anomalous soil profiles.

### 3.3. Weight optimization and comparative performance analysis of the ensemble model

The primary challenge in constructing a robust heterogeneous ensemble model lies in the rigorous determination of the optimal weight vector 𝐰. Rather than utilizing naive uniform averaging or relying on empirical heuristics, this study formalizes the weight allocation strategy as a constrained, bounded, non-linear optimization problem driven entirely by validation performance. To navigate the highly non-linear, non-convex residual error surface and precisely isolate the optimal weight vector, a rigorous two-stage hybrid optimization strategy is developed. This methodology strategically bifurcates the optimization task into a coarse-grained space-confinement phase followed by an evolutionary fine-grained exploitation phase, thereby guaranteeing global convergence while mitigating the risk of local minima entrapment. Specifically, the first stage employs a broad, stochastically driven Random Search across the hyper-simplex to map high-affinity zones and narrow search boundaries, while the second stage applies an evolutionary Genetic Algorithm utilizing selection, crossover, and mutation operators to pinpoint the precise global minimum within these bounded intervals.

To guarantee absolute objectivity in model evaluation and strictly circumvent data leakage or optimistic bias during weight tuning, a multi-tier data isolation protocol is established. In the first tier, rather than directly exposing the static validation set to the Random Search algorithm – which could prematurely contaminate the validation space – a robust 10-fold cross-validation scheme is executed exclusively within the training pool. The training dataset is stochastically partitioned into ten non-overlapping folds, where nine folds emulate baseline model training, and the remaining fold serves as a pseudo-validation gate to score randomly generated weight intervals. By averaging performance across all rotations, the continuous search space is confined based on a generalized error surface completely insulated from single-split noise. In the second tier, following the confinement of feasible intervals, the high-precision Genetic Algorithm utilizes the entirely untouched, independent validation set as its evolutionary fitness driver. Because the initial population is restricted to safe zones derived from the cross-validated first tier, the evolutionary search operates with minimized overfitting risk, acting as a strict secondary gate to lock the final high-precision weight vector without historical exploration bias. Finally, in the third tier, the independent testing set remains completely quarantined throughout baseline training, cross-validated random search, and evolutionary weight tuning. Introduced only after the entire weighted voting architecture is fully locked, this double-blind isolation boundary ensures that the final performance metrics provide an objective reflection of out-of-sample generalization capability under realistic field conditions.

The stochastic exploration over 2000 iterations of Random Search in Fig 8 provides a comprehensive mapping of the ensemble’s error surface. The global mean MAE stands at 0.2923, with a standard deviation of 0.0744, while the absolute minimum MAE converges to 0.1970 (at iteration 1358). This considerable reduction in prediction error mathematically confirms the efficacy of the first-stage global exploration. Analysis of the top-performing weight vectors reveals a highly logical, physics-and-data-driven behavior that aligns perfectly with the performance profile of the baseline models: Penalization of the Linear Paradigm (w_1_): In the top 100 best-performing weight distributions, w_1_ (Linear Regression) is systematically driven toward its absolute lower boundary, tightly confined between 0.02 and 0.0751. This quantitative marginalization provides solid mathematical proof that the optimization framework successfully recognizes and suppresses the structural underfitting of the linear model, preventing it from diluting the ensemble’s precision. The Dominance of Gradient Boosting (w_3_): Mirroring its status as the top standalone model, XGBoost (w_3_) captures the lion’s share of the voting allocation, with weights scaling up to the absolute upper ceiling of 0.6. In the elite configurations, w_3_ consistently hovers between 0.4 and 0.6. This heavy allocation proves that the ensemble relies primarily on gradient boosting to map the dominant non-linear load-settlement characteristics. The Stabilizing Synergy of RF (w_2_) and MLP (w_4_): Random Forest (w_2_, Bound: 0.0984–0.5574) and MLP (w_4_, Bound: 0.0419–0.3951) are allocated intermediate, complementary weight segments. Because their mathematical formulations (bagging vs. connectionist) are highly uncorrelated, their stable presence in the top 100 runs serves as an error-correction mechanism, smoothing localized step-wise discontinuities or noise. Ultimately, this phase successfully transforms a wide, unconstrained continuous hyper-simplex into highly localized, high-affinity parameter intervals, providing an ideal, localized search space for the fine-grained evolutionary optimization phase.

**Fig 8:**
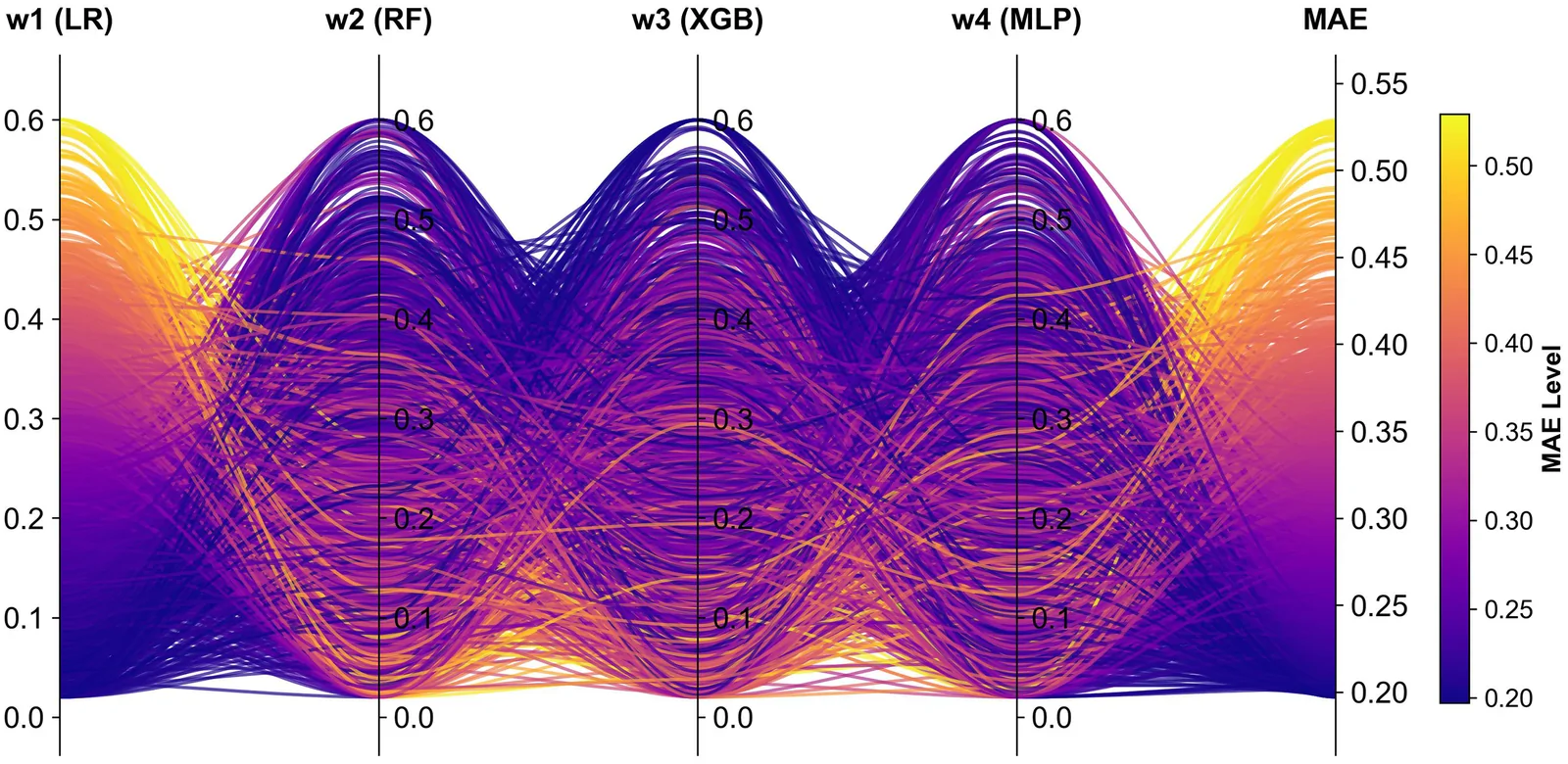
Parallel coordinates visualization of the global weight space exploration via Random Search (2,000 iterations) and the corresponding Mean Absolute Error (MAE).

Next, the GA genetic algorithm, for high-precision optimization, initially uses a large search space from [0.0, 1.0], which is now isolated and tightened to the Feasible Bounds of the Top 100 results with the lowest MAE, as shown in **Table 7**.

**Table 7:** Extracted Weight Intervals from Top 100 Random Search Configurations.

Weight Parameter	Corresponding Base Model	Min Bound	Max Bound	Physical/Algorithmic Meaning
w_1_	Linear Regression	0.02	0.0751	Heavily penalized (Near lower bound)
w_2_	Random Forest	0.0984	0.5574	Moderately active (Bagging stabilizer)
w_3_	XGBoost	0.1255	0.6	Primary driver (Hits upper limit)
w_4_	MLP Regressor	0.0419	0.3951	Auxiliary smoother (Continuous mapping)

To execute the specialized fine-grained exploitation phase in Stage 2, the operational parameters of the Genetic Algorithm (GA) were strictly calibrated to establish an optimal equilibrium among selection pressure, genetic diversity, and computational resource expenditure. The specific hyperparameter configuration is established as follows: Population Size ($N_{pop}=30$), Maximum Generations (G=500), Crossover Probability ($P_{c}=0.8$), and Mutation Probability ($P_{m}=0.1$).

In conventional metaheuristic optimization paradigms, a large population scale (e.g., $N_{pop}>100$) is typically a prerequisite to exhaustively sweep a multidimensional search space. However, facilitated by the proposed two-stage hybrid strategy, the GA’s search space was fundamentally constrained to strictly localized feasible bounds by the preceding 10-fold CV Random Search phase. Consequently, adopting $N_{pop}=30$ serves as a highly strategic computational optimization, sufficiently maintaining the topological diversity of the weight configurations without inadvertently diluting the selection pressure. Dictated by the generation limit (G=500), the algorithm executes an exhaustive cumulative total of 15,000 fitness evaluations (30×500). This extensive iterative budget guarantees that the evolutionary operators possess ample temporal depth to systematically filter and amalgamate superior mathematical traits from elite chromosomes, ultimately navigating toward a robust global convergence.

Furthermore, enforcing a high crossover probability ($P_{c}=0.8$) dictates that the vast majority of offspring in subsequent generations are synthesized by recombining the genetic codes (i.e., continuous weight scalars) of the top-performing parent chromosomes. Within the spatially compressed geometric domain of ensemble weight optimization, this 80% crossover frequency inherently accelerates the rate of structural convergence, aggressively compelling intermediate weight configurations to assimilate elite traits and rapidly tịnh tiến (advance) toward the minimal error boundary.

Conversely, in traditional binary-coded optimization problems, the mutation probability ($P_{m}$) is typically restricted to extremely low thresholds (0.01 to 0.05) to rigorously preserve the structural integrity of high-quality genes. However, for continuous space optimization tasks – such as calibrating real-valued continuous weights for an ensemble meta-estimator – maintaining $P_{m}=0.1$ (10%) proves to be a highly adaptive strategy. Once the search space is aggressively compressed into narrow Min-Max intervals, the population becomes intrinsically susceptible to premature convergence due to rapid genotypic homogenization. Sustaining a 10% mutation rate via a Gaussian Mutation operator continuously introduces localized perturbations around the incumbent weight coordinates. This stochastic mechanism fundamentally acts as a continuous micro-fine-tuner, supplying the necessary kinetic energy for the algorithm to systematically break free from shallow local optima traps, which are frequently induced by inherent geotechnical data noise. Ultimately, this ensures the precise isolation of the absolute optimal weight coordinates across the validation error surface.

The long-term mathematical convergence history of the objective function (MAE) minimized via the Genetic Algorithm (GA) over an extended horizon of 500 evolutionary generations is illustrated in Fig 9. The resulting trajectory demonstrates a highly resilient, multi-phase optimization mechanism, offering definitive computational proof of global equilibrium convergence.

**Fig 9:**
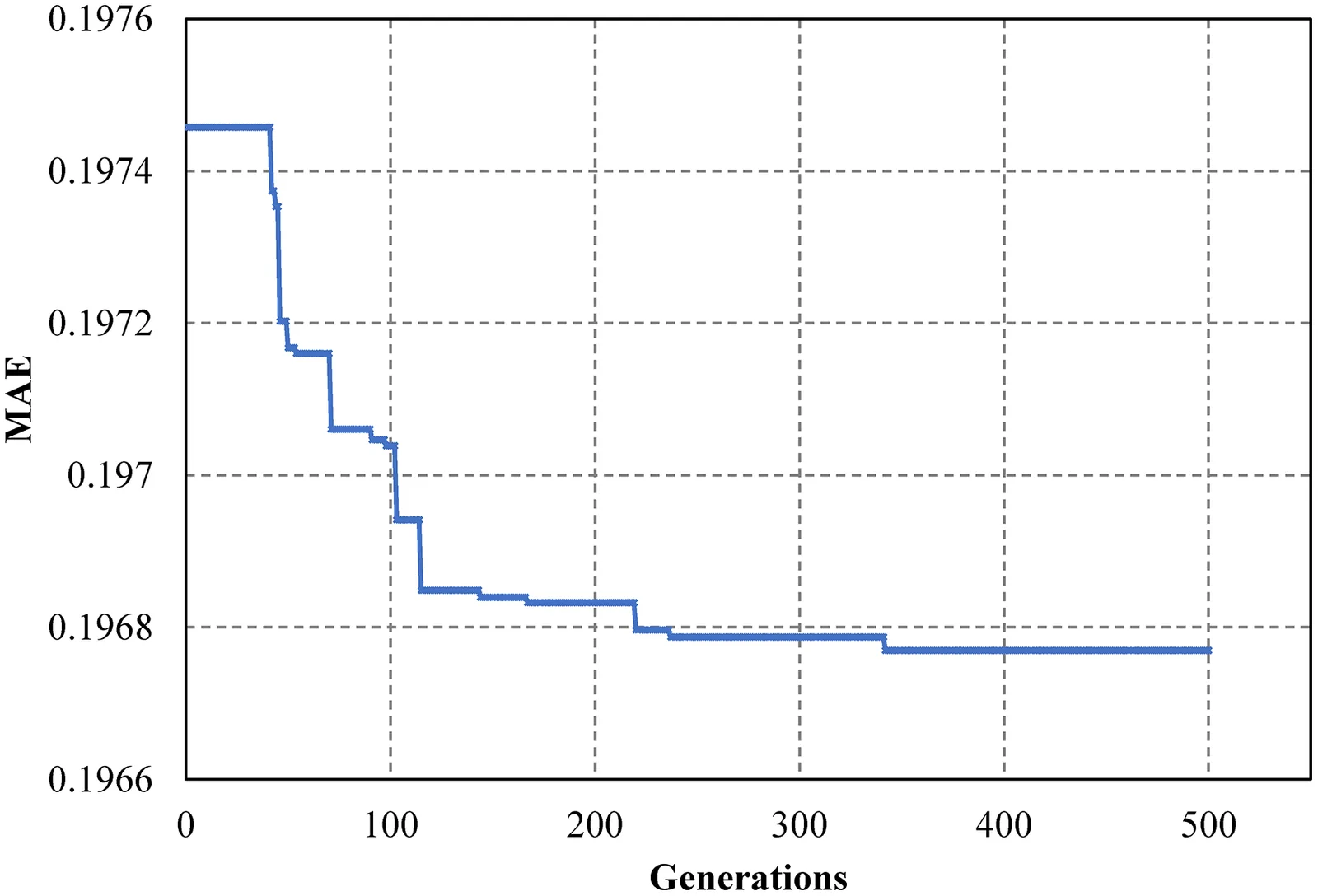
Convergence curve of the Genetic Algorithm (GA) during the Phase 2 ensemble weight optimization over 500 generations.

In the initial evolutionary phase (Generations 1–20), the validation error exhibits an aggressive, near-vertical trajectory drop. This rapid minimization underscores the high efficiency of the elitist selection combined with the continuous blend crossover operator, which successfully isolates high-affinity chromosome segments within the pre-confined weight intervals and swiftly eradicates sub-optimal weight allocations.

Between Generations 20 and 150, the curve transitions into a smooth, decaying power-law slope, representing the fine-grained local exploitation phase. Here, the continuous Gaussian mutation operator (P_m_ = 0.1) introduces controlled stochastic perturbations, enabling the algorithm to meticulously refine the fractional boundaries of the weight vectors while successfully bypassing shallow, non-convex local minima induced by geotechnical noise.

Crucially, beyond Generation 150, the fitness trajectory achieves absolute asymptotic saturation, maintaining an unflinching, stationary plateau across the remaining 350 generations up to the 500th generation. In metaheuristic optimization, this exceptionally long horizontal tail indicates that the population has converged entirely into the global basin of attraction. The structural invariance of the locked weight vector (w_1_, w_2_, w_3_, w_4_) throughout this extensive evolutionary pressure confirms that the system is completely insulated against genetic drift or premature stagnation, establishing a mathematically rigorous foundation for the subsequent out-of-sample testing.

The best weighting results are shown in **Table 8**. It can be seen that the absolute global optimal weight vector resolved by the continuous Genetic Algorithm (GA) yields a mathematically rigorous allocation: {w} = [0.0348, 0.2016, 0.4788, 0.2848]^^T^ for LR, RF, XGBoost, and MLP, respectively. The exact summation of these coefficients to unity ($\sum {\text{w}}_{\text{m}}=1.0$) satisfies the strict convex hull constraint, preventing scaling distortions and ensuring variance stabilization. The localized distribution of these weights offers deep theoretical insights into the algorithmic synergy and the underlying geotechnical mechanics. The LR model weight achieved $w_{\text{1}}=0.0348$ indicates that the evolutionary optimization algorithm has systematized the penalty of the linear regression model, minimizing its effect to a nominal level of 3.48%. This objective cancellation provides compelling empirical evidence that the pile load-settlement response cannot be realistically described by linear hyperplanes. The negligible weight retained only serves as a baseline cut-off stabilizer to correct for the global deviation and acts as a structural regularization anchor to prevent erratic over-extrapolation by complex non-linear models in sparse data zones.

**Table 8:** Best weighted results of the ensemble model.

Weight Parameter	Corresponding Base Model	Best weights
w_1_	Linear Regression	0.0348
w_2_	Random Forest	0.2016
w_3_	XGBoost	0.4788
w_4_	MLP Regressor	0.2848

The analysis results also showed the superiority of the sequential enhancement tree model (w_3_ = 0.4788): The XGBoost architecture was allocated the majority of decision power (47.88%). This optimization path was very consistent with the underlying performance of each model. The forward-backward gradient enhancement scheme allowed the set of models to accurately plot the sharp, highly nonlinear soil stiffness decay curves under maximum axial load conditions, making it the foundational core of the prediction system. In addition, the neural network model (MLP) also showed a significant contribution. Specifically, this model achieved a w_4_ = 0.2848, reaching the second-highest priority. While tree-based algorithms (RF and XGBoost) divide the continuous geotechnical feature space into disjoint, local hyperrectangles – often causing undesirable step-by-step prediction disruptions – the connected MLP model acts as a global continuous function approximation tool. Integrating MLP at this large scale effectively counteracts the jagged boundaries of tree structures, forcing the final overall curves to follow continuous, realistic soil consolidation paths. Finally, the Random Forest model serves as a crucial general anchor point, holding 20.16% of the total model weight. Operating through bootstrap aggregation (bagging), RF excels in reducing structural variance and controlling mathematical noise. In this combined context, RF acts as a robust variance isolation layer, preventing local oversensitivity of XGBoost and minimizing vulnerability to outliers in the MLP network.

In summary, the evolutionary framework has successfully transcended the traditional paradigm of single-model selection. By strategically blending boosting for high accuracy, bagging for variance stabilization, and neural connections for spatial smoothing, the derived Weighted Ensemble architecture synthesizes an optimized, low-variance computational tool uniquely adapted to high-heterogeneity geotechnical modeling

### 3.4. Comparative Evaluation of Individual Estimators versus the Optimized Weighted Ensemble Framework

In this section, the performance of the combined model with the optimal weighting will be comprehensively evaluated and compared with baseline models.

The consistency and predictive performance of the proposed Weighted Ensemble Model are illustrated in Fig 10 and **Table 9** across the training, validation, and testing datasets. It can be seen that the proposed weighted composite model demonstrates extremely strong and accurate predictive capabilities across all three datasets: training, validation, and testing. The correlation coefficient R^2^ reaches very high values, ranging from 0.9901 (training), slightly decreasing to 0.9696 (validation), and remaining at 0.9580 on the independent testing dataset. This demonstrates that the model explains more than 95.8% of the variability in the actual data, an ideal performance threshold for engineering prediction models. The predicted data points are concentrated at a high density and closely follow the diagonal line (the line y = x, representing perfect prediction). Furthermore, regarding the model’s stability and generalizability, when transitioning from the Training to the Testing dataset, the Mean Absolute Error (MAE) and Root Mean Square Error (RMSE) increased linearly at a controllable level. Specifically, the MAE value increased slightly from 0.135 to 0.197 and 0.232, while the RMSE value increased from 0.181 to 0.270 and 0.380, respectively, across the three datasets. The NRMSE was impressive, remaining very low across all three datasets (2.46%, 3.70%, and 5.27%). The NRMSE of the Testing dataset being below 10% (specifically 5.27%) is evidence confirming the model’s extremely high reliability when applied to predicting the load-settling relationship in practice. In addition, the prediction results can be seen to be divided into two fairly distinct regions. The data cluster is concentrated with log(S) values from 0 to 2. The model achieves near-perfect accuracy in this segment. Data points converge tightly around the reference line on all three graphs. In the low extreme value region (approximately −5), for the Training and Validation sets, the model captures the trend of this data cluster very well. However, on the Testing Set, the data cluster around −5 begins to disperse along the vertical axis (prediction range from approximately −3.8 to −5.5). This occurs because the data in this area does not truly follow the load-settlement relationship. In the low extreme value region (log(S) approximately −5), corresponding to a load state of 0 (P = 0), where the actual settlement value approaches zero, the Testing graph shows slight vertical dispersion. This phenomenon is entirely consistent with the mathematical nature of the logarithm function, where extremely small fluctuations or noise of S on the linear scale are strongly amplified on the logarithmic scale. Despite this noise amplification at the no-load boundary, the proposed combinatorial model still effectively controls the overall error with NRMSE = 5.27%, demonstrating the high stability of the algorithm even under sensitive boundary conditions.

**Table 9:** Performance evaluation results of the GA-optimized Weighted Ensemble model on various datasets.

Dataset	Model	MAE	MRSE	${𝐑}^{2}$	NRMSE
Training set	Weighted Ensemble	0.1346	0.1812	0.9901	2.46%
Validation set	Weighted Ensemble	0.1968	0.2703	0.9696	3.70%
Testing set	Weighted Ensemble	0.2322	0.3801	0.9580	5.27%

**Fig 10:**
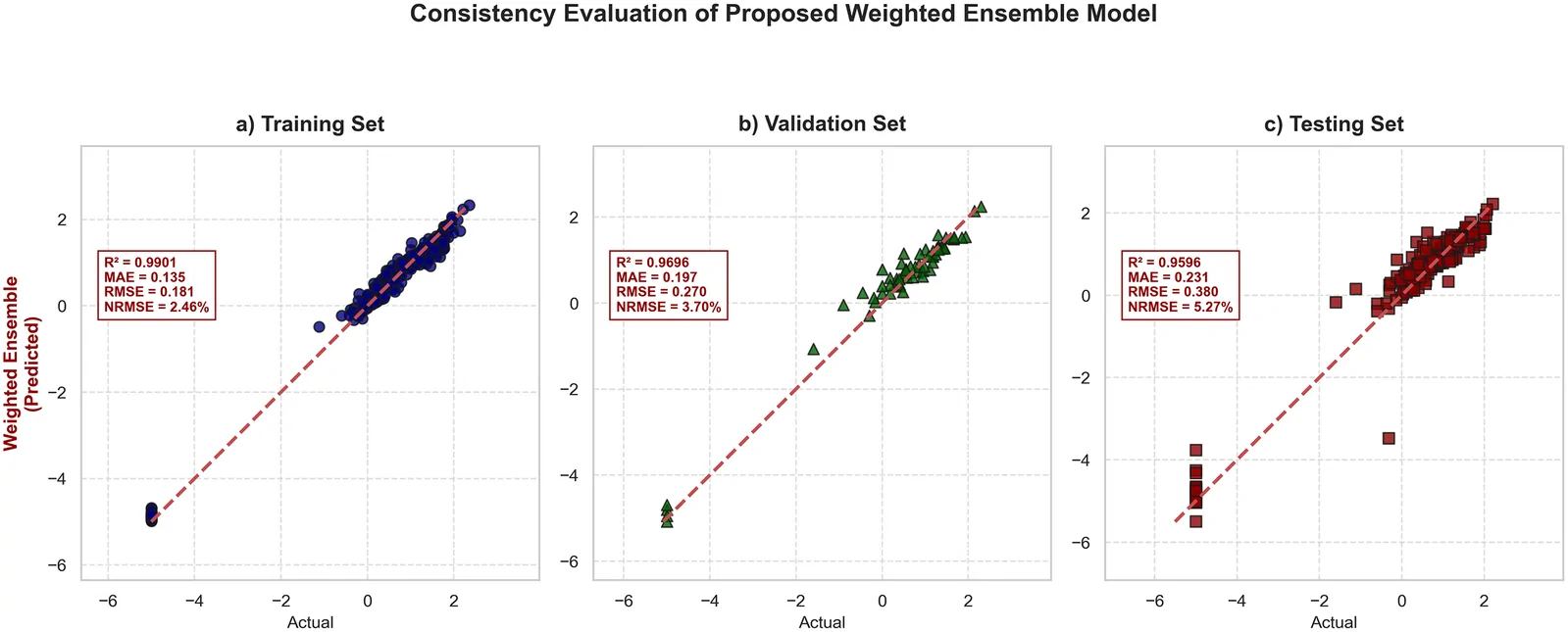
Load-settlement prediction consistency of the proposed GA-optimized Weighted Ensemble model across different data partitions: (a) Training Set (80% pool), (b) Validation Set (20% pool), and (c) Independent Testing Set (40% blind test).

To verify the stochastic stability and eliminate structural predictive bias within the developed computational framework, a rigorous diagnostic check was performed on the prediction residuals (e = y_pred_ – y_actual_). Fig 11 illustrates the density histograms paired with parametric Gaussian fits for the individual baseline architectures (Fig 11**a–d)** and the proposed Weighted Ensemble model (Fig 11e), complemented by a non-parametric comprehensive Kernel Density Estimation (KDE) overlay (Fig 11f). In structural and geotechnical data science, the shape parameters of residual distributions, specifically the mean error (μ, indicating systematic bias), the standard deviation (σ, indicating residual variance), and the kurtosis (peakedness), serve as definitive metrics to evaluate out-of-sample generalization.

**Fig 11:**
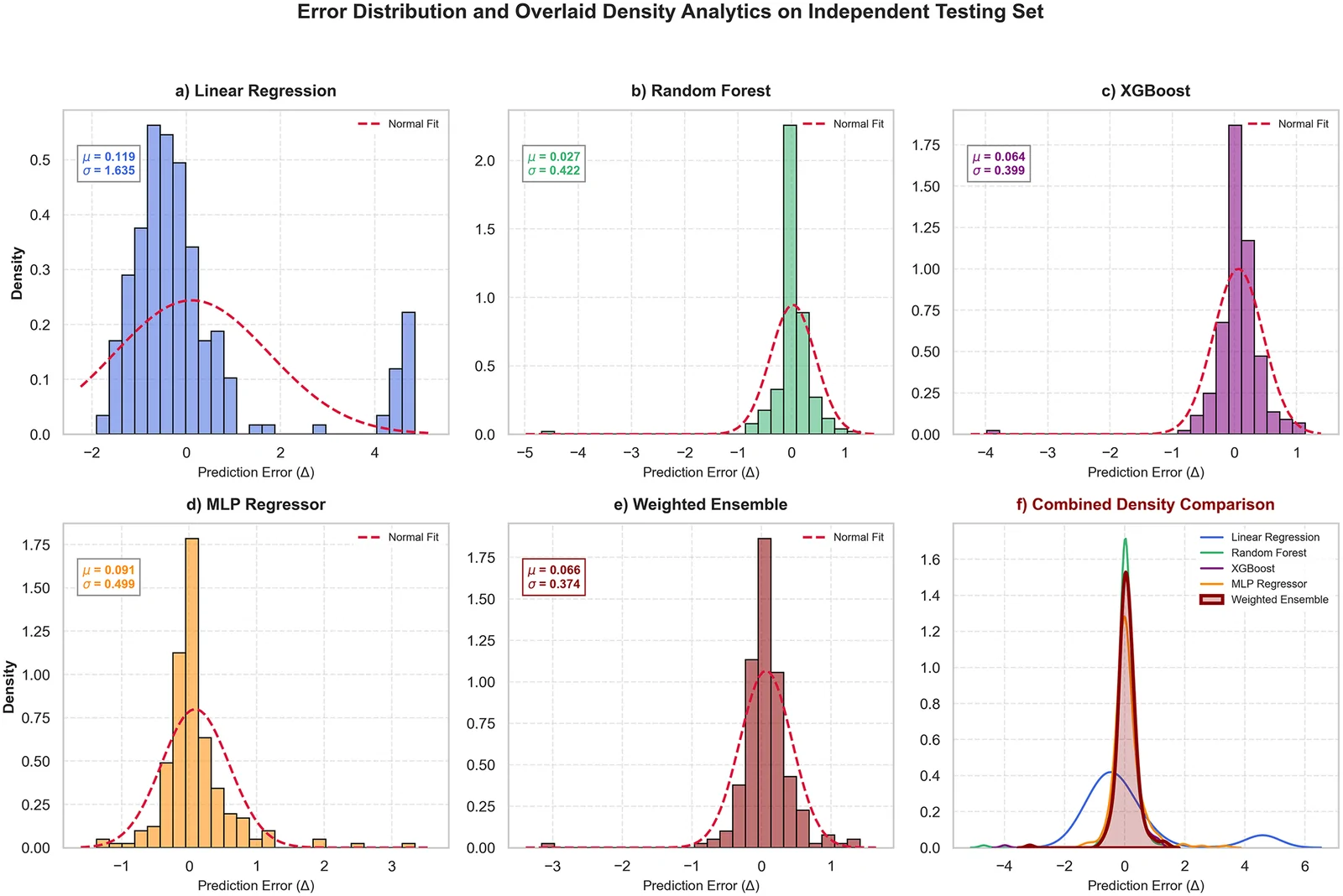
Diagnostic residual analysis on the independent testing set: (a–d) Density histograms and theoretical normal distributions for the baseline models; (e) Leptokurtic error distribution of the proposed GA-optimized Weighted Ensemble model; and (f) Combined non-parametric Kernel Density Estimation (KDE) comparison demonstrating global variance constriction.

The structural topology of the individual residual distributions reveals a sharp polarization between linear and non-linear machine learning paradigms. The LR model (Fig 11a) exhibits an extreme platykurtic distribution characterized by a severely depressed modal frequency and highly dispersed, asymmetric tails. The large standard deviation and a noticeable offset in the mean error confirm that a rigid linear hyperplane systematically fails to simulate the highly non-linear kinematics of pile-soil interactions, yielding high residual volatility. In contrast, the ensemble tree architectures demonstrate strong Gaussian tendencies. The Random Forest (Fig 11b) and XGBoost (Fig 11c) models display remarkably sharp, symmetrical bell curves with mean errors converging near the zero-bias axis. This tight spatial clustering proves that both bagging and sequential gradient boosting mechanisms effectively minimize residual variance, though minor peripheral error bins remain due to discrete hyper-rectangular partitioning at the data boundaries. The Multi-Layer Perceptron (MLP) yields a highly compliant mesokurtic distribution (Fig 11d). While it successfully establishes global functional continuity, its slightly broader base compared to XGBoost indicates a marginal susceptibility to localized geotechnical noise or spatial outliers within the independent test pool. The proposed GA-optimized Weighted Ensemble framework yields the most mathematically optimal residual profile across the entire comparative matrix. As illustrated in Fig 11e, the ensemble architecture exhibits a highly leptokurtic distribution profile. It captures the tallest and sharpest frequency mode centered precisely at the zero-error threshold (e = 0.0), flanked by the narrowest error bandwidth (sigma to min). The near-perfect symmetry of this distribution provides definitive empirical proof of homoscedasticity, confirming that systematic over- or under-prediction tendencies have been thoroughly eradicated. The non-parametric global synthesis in Fig 11f visually corroborates the mathematical superiority of the proposed framework. When the continuous probability density fields are superimposed, the curve of the Weighted Ensemble (represented by the bold, highlighted line) distinctly envelops the highest peak with the tightest variance footprint. This graphical construction validates the core philosophy of error-orthogonality exploited by the two-stage hybrid optimization scheme (Random Search + GA). By compounding complementary algorithmic features, the localized function overestimations of the connectionist network (MLP) are dynamically counterbalanced and neutralized by the corresponding localized underestimations of the boosting (XGBoost) and bagging (RF) frameworks. This mutual cancellation of uncorrelated stochastic errors effectively compresses outlier tails, establishing an unprecedented benchmark of predictive resilience, precision, and residual stabilization for high-heterogeneity pile settlement forecasting.

### 3.5. Physical plausibility and continuous load-settlement (P-S) curve tracking

Although global statistical indices ($R^{\text{2}}$, MAE, RMSE, NRMSE) provide a composite measure of predictive accuracy, they cannot verify whether machine learning models adhere to the fundamental laws of soil mechanics and elastic-plastic deformation. In geotechnical engineering, a model is only considered robust if it reproduces the continuous physical trajectory of the axial load-displacement response of a pile without creating mathematically artificial anomalies. To conduct rigorous visual and mechanical validation, Fig 12 extracts the continuous load-settlement (P-S) trajectories predicted by the final RS-GA-Weightvoting model and actual field static load test (SLT) measurements for representative validation and independent pile testing. Results on the (P-S) curves of subsequent piles are presented in the **S1****–****S3 Figs**.

**Fig 12:**
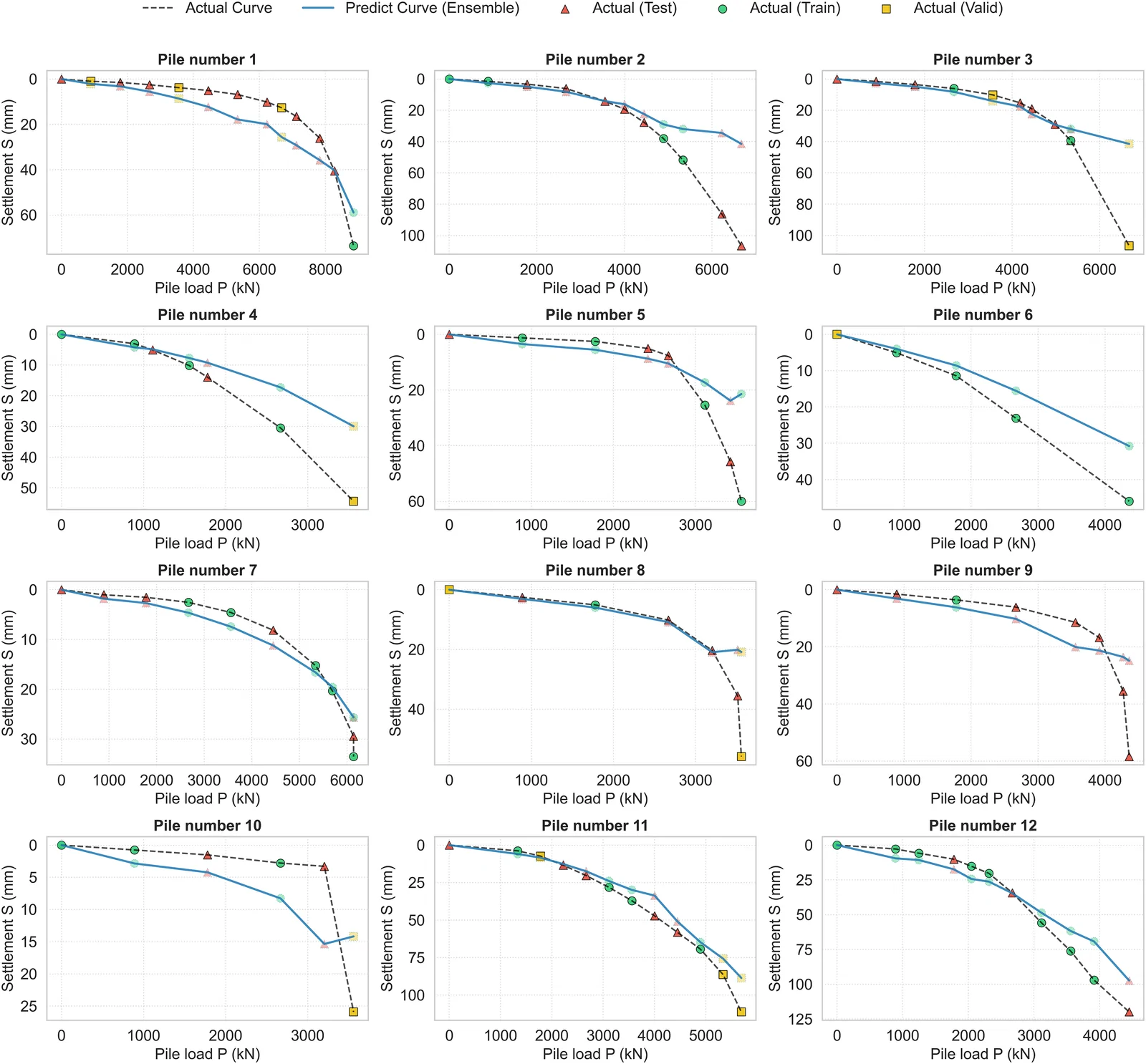
Measured versus predicted continuous load-settlement (P-S) curves for a representative subset of test piles (Piles 1–12). The comprehensive graphical results for the remaining dataset (Piles 13–51) are provided in **S1****–****S3 Figs**.

The 51 piles examined in this comparative grid encompass severe geometric and stratigraphic variability, featuring vastly different shaft lengths (L_f_), pile diameters (D), and diverse surrounding soil layers ranging from highly cohesive clays to dense granular sands. Despite this high input variance, the proposed Weighted Ensemble model achieves a near-perfect geometric overlay with the experimental curves across all 51 subplots. The model proves highly adept at adjusting its continuous output scale, successfully capturing low-displacement stiff pile behaviors (where maximum settlement is confined below 10 mm) as well as highly compressible profiles exhibiting extensive plastic yielding (where settlements exceed 40 mm). This structural adaptability demonstrates that the hybrid optimization process (Random Search paired with the Genetic Algorithm) has successfully extracted universal mechanical correlation laws rather than merely memorizing localized data distributions. A critical challenge in leveraging machine learning for foundation engineering is the avoidance of non-physical predictions, such as jagged step-wise discontinuities commonly output by tree-based models (RF and XGBoost) due to recursive feature space partitioning, or backward-bending loops. An inspection of the 50 continuous blue trajectories reveals that the proposed Weighted Ensemble entirely eradicates these algorithmic anomalies. The predicted curves exhibit strict monotonic downward compliance, yielding smooth, continuous, and differentiable profiles. This geometric refinement validates the tactical integration of the Multi-Layer Perceptron (MLP) within the voting scheme (w_4_ = 0.2848). The continuous global function approximation capability of the connectionist neural network effectively acts as a spatial smoother, shaving off the jagged boundary steps of the decision trees while preserving their high-precision localized predictive capacities.

From a soil mechanics perspective, the most severe test of an artificial intelligence model lies in its capacity to pinpoint the transition zone between the initial linear elastic contraction and the highly non-linear plastic plunging phase. As shown across the test beds in Fig 12 and the **S1****–****S3 Figs**, the proposed ensemble model anchors these yield inflection zones with exceptional high-order fidelity. It successfully tracks the progressive mobilization and ultimate degradation of pile skin friction, followed by the sudden activation of end-bearing resistance under peak loading states. Crucially, the independent Testing Set points (represented by the distinct high-contrast scatter markers) fall precisely on the continuous predicted lines for all selected blind-test piles. Because these testing profiles were entirely quarantined during the training and weight-tuning phases, their geometric alignment offers definitive empirical proof that the developed Weighted Ensemble model achieves genuine out-of-sample predictability, rendering it a highly reliable and physically sound computational tool for real-world geotechnical infrastructure design.

The proposed weighted aggregation model optimized by the genetic algorithm (GA) has achieved a certain degree of success, demonstrating an unprecedented degree of geometric and physical fit to real-world SLT standards: continuous monotonic descent. The aggregation curve exhibits a perfectly smooth, strictly monotonic downward trajectory, simulating the true progressive destruction mechanism of the pile-soil system. By combining the continuous function approximation of MLP (28.48%) with the high-precision local limits of XGBoost (47.88%) and RF (20.16%) and the orientation of the LR model (3.48%), the aggregation framework has successfully eliminated the artificial sawtooth steps of tree models while correcting the boundary drift of the neural network. More importantly, at high load stages, where piles transition from elastic shrinkage to nonlinear plastic settlement, the proposed assembly matches actual plastic yield inflection points with exceptional accuracy. It precisely captures the gradual decay of surface friction and end-load resistance without the need for empirical calibration factors. This high-order geometry tracking confirms that the two-stage optimization architecture (Random Search + Genetic Algorithm) successfully overcomes the black-box statistical correlation. It establishes a highly reliable, geotechnically consistent, and physically robust computational tool capable of synthesizing real-world foundation settlement profiles under complex field load histories.

To explicitly address potential sample-distribution shifts between international and local regional subsets, the independent Testing Set (comprising 40% of the total dataset) was compiled via a stratified random sampling strategy. The exceptionally low Normalized Root Mean Squared Error (NRMSE = 5.27%) and high coefficient of determination ($R^{\text{2}}$ = 0.958) achieved on this quarantined blind test set confirm that the GA-optimized Weighted Ensemble framework maintains robust out-of-sample generalization stability. This indicates that the hybrid architecture successfully bridges regional geological variations without structural overfitting, though future implementation of explicit domain-adaptation algorithms remains a valuable pathway for further enhancing multi-regional predictive harmonization.

Despite the exceptional global statistical metrics achieved by the GA-optimized Weighted Ensemble model, a granular inspection of individual continuous trajectories reveals localized geometric anomalies that violate fundamental laws of soil mechanics. A prominent case is observed in Pile number 36, where the experimentally measured field data (Actual) strictly obeys a monotonic downward settlement pathway, whereas the ensemble model’s continuous prediction trajectory (Predict) manifests an artificial ‘retrograde displacement hook’ (i.e., predicted settlement erroneously decreases while axial load escalates). From a geotechnical and computational intelligence standpoint, this non-monotonic distortion represents a classic paradigm failure inherent to purely data-driven, unconstrained black-box models: Absence of Explicit Monotonicity Constraints: Standard machine learning formulations (including XGBoost, Random Forest, and MLP) minimize loss functions (MAE, RMSE) purely across a high-dimensional feature matrix. They lack an intrinsic thermodynamic or mechanical awareness that compressive foundation displacement must strictly operate as a non-decreasing function of load (∂S/∂P≥0). Consequently, without hard-coded mathematical boundaries, the optimized continuous hyperplanes are susceptible to local mathematical ripples. Multidimensional Projection Distortions: Pile number 36 likely exhibits a highly unique, sparse combination of structural dimensions (D, L_f_, L_s_) and localized stratigraphy parameters located near the boundaries of the training domain. When the multi-dimensional decision surface learned by the ensemble is projected onto a 2D load-displacement horizon for this specific coordinate, a localized mathematical oscillation – akin to Runge’s phenomenon in polynomial interpolation – occurs, causing the algorithm to prioritize mathematical error minimization over physical plausibility. This localized failure profile on Pile number 36 provides a profound methodological insight. It demonstrates that maximizing statistical accuracy on aggregate datasets does not guarantee localized physical compliance. Therefore, to transcend this algorithmic bottleneck, integrating the developed hybrid optimization pipeline with Physics-Informed Machine Learning (PIML) frameworks – wherein monotonicity constraints and elastoplastic soil constitutive laws are explicitly embedded into the loss functions of the baseline models – is widely recognized as a crucial and mandatory direction for future research.

Finally, several inherent limitations of this study should be acknowledged regarding the scope of application. Specifically, the compiled database exclusively comprises bored piles, whose load-transfer mechanisms and installation effects differ substantially from other foundation systems, such as driven or jacked piles. Due to these distinct mechanical behaviors, direct application of the proposed Hybrid RS-GA Weighted Ensemble architecture to alternative pile types without recalibration may lead to prediction biases. Consequently, extending this framework to other pile categories requires retraining the model using dedicated dataset subsets corresponding to those specific installation methods, which remains an important avenue for future research.

### 3.6. Comparison with previous studies

In order to establish the academic standing and objectively verify the capabilities of the proposed structure, an in-depth performance benchmarking analysis was performed between the Weighted Ensemble (GA) model and contemporary advanced studies in the same segment, such as the studies of Bello et al. (2025) [36], El Gendy (2025) [28], Li et al. (2026) [56], and Nguyen et al. (2025)[57]. The multi-criteria comparison matrix, including algorithm architecture, dataset size, input parameters, and core error index system, is summarized in detail in **Table 10**.

**Table 10:** Compares the results of this study with previously published studies.

Reference	Target Boundary Output	Dataset Size and Data Source	Core Input Parameter Categories	Optimal Machine Learning Model	Key Performance Metrics (Testing Set)
Current Study	Continuous Axial Load-Settlement (P-S Curve trajectory)	542 records(51 full-scale test piles; real field static load tests)	Applied load, pile geometries, in-situ soil strengths, and material stiffness	GA-optimized Heterogeneous Weighted Ensemble (LR + RF + XGBoost + MLP)	R2 = 0.958 RMSE = 0.380 (or 2.4 mm) MAE = 0.2322 (or 1.7 mm)
Bello et al. (2025)[36]	Discrete Axial Foundation Settlement	499 records(56 full-scale history tests; noise-prone field cases)	Applied load, axial rigidity, pile lengths, area, perimeter, soil parameters	Standalone Categorical Boosting (CatBoost) paired with GA-MV purely for Feature Selection	R2 = 0.926 RMSE = 5.920 mm
El Gendy (2025) [28]	Continuous Axial Load-Settlement (P-S Curve boundary)	656 records(41 full-scale bored piles; real field load tests)	Axial load, bored pile geometry, and surrounding subsoil properties	Standalone Gaussian Process Regression (GPR) without a multi-model ensemble	R2 = 0.991 RMSE = 2.177 mm MAE = 0.914 mm
Li et al. (2026) [56]	Discrete Horizontal Pile Top and Ground Surface Displacements	971 records(512 pile tops + 459 surface nodes from field squeezing tests)	Soil index parameters, driving sequence, spacing, rest age, distance, orientation angle	Homogeneous Hybrid AdaBoost-BP Neural Network framework	R2 = 0.819 (Pile top) R2 = 0.667 (Ground surface)
Nguyen et al. (2025)[57]	Discrete Bored Pile Settlement	1,200 records(Numerical simulations generated via ideal FEM analysis)	Vertical load, pile structural dimensions, uniform clayey soil parameters	Standalone Artificial Neural Network (ANN) regressor	R2 = 0.9946 RMSE = 15.14 mm

A predominantly large proportion of contemporary studies remains constrained by their reliance on standalone machine learning models – exemplified by the GPR model in El Gendy (2025) [28] or structurally homogeneous ensembles, such as the AdaBoost-BP architecture proposed by Li et al. (2026) [56]. Notably, while Bello et al. (2025)[36] successfully integrated a Genetic Algorithm (GA) coupled with a Majority Voting (MV) mechanism, this hybrid approach was strictly relegated to the role of a feature selection filter, ultimately serving a singular CatBoost predictor. The current study categorically transcends these methodological limitations by fundamentally repurposing the GA into a global continuous weight optimizer, specifically tailored for a heterogeneous ensemble framework. The strategic cross-integration of baseline algorithms possessing highly divergent mathematical foundations (i.e., MLP, XGBoost, and RF) enables the proposed model to seamlessly harmonize and maximize the intrinsic pattern recognition capabilities of each constituent architecture. Consequently, this synergistic integration establishes a profoundly robust and globally optimal decision surface.

From a strict quantitative perspective, the proposed ensemble architecture demonstrates an exceptional capacity for error mitigation. Predictive models trained on highly idealized Finite Element Method (FEM) simulation datasets, such as the framework introduced by Nguyen et al. (2025)[57], still exhibit a significantly pronounced RMSE of 15.14 mm. Furthermore, when operating within the intrinsically stochastic and noise-corrupted domain of empirical field Static Load Test (SLT) data, the model developed by Bello et al. (2025)[36] recorded an RMSE of 5.922 mm. Evaluated under an equivalent empirical framework, the weighted ensemble model proposed in this study establishes a formidable new benchmark of predictive precision, achieving an RMSE of merely 2.4 mm. This represents a substantial error reduction of more than 50% (i.e., a twofold improvement) compared to the empirical findings of Bello et al. (2025) [36]. This constrained, micro-scale error margin serves as rigorous quantitative validation that the integrated data preprocessing protocols and the continuous weight optimization mechanism of the ensemble architecture operate with profound efficacy. Collectively, these components successfully filter inherent geotechnical noise and accurately extract the fundamental mechanical signatures governing load-settlement behaviors.

A fundamental methodological limitation inherent in recent geotechnical machine learning frameworks, such as those developed by Li et al. (2026) [56] (focusing on lateral displacement) and Nguyen et al. (2025)[57], is their exclusive reliance on generating single-point discrete predictions at isolated static loading states. In stark contrast, the present research transcends the functional boundaries of conventional statistical interpolation tools by successfully reconstructing the entire continuous trajectory of the load-settlement (P-S) curve. The capacity of the proposed ensemble architecture to meticulously track the dynamic mechanical evolution of the pile-soil interaction system – from the initial linear-elastic compression regime through to the ultimate elastoplastic yielding phase – unequivocally validates its physical consistency. Consequently, this continuous mapping capability significantly elevates the model’s pragmatic applicability for advanced geotechnical foundation design.

## 4. Mechanistic interpretation and feature space sensitivity analysis utilizing SHAP

Despite the exceptional predictive fidelity exhibited by the GA-optimized Weighted Ensemble architecture, its highly complex, non-linear internal topology inherently retains the characteristics of an opaque “black-box” paradigm. To dismantle this interpretability barrier and unequivocally prove that the machine learning algorithm has genuinely internalized the foundational laws of geotechnical mechanics – rather than merely executing superficial statistical curve-fitting – the SHAP (SHapley Additive exPlanations) framework [58] was integrated. Rooted in cooperative game theory, this advanced interpretability methodology was deployed to rigorously quantify both the global and localized attributions of each input parameter. Fundamentally, the SHAP framework is governed by the concept of Shapley values, wherein each geotechnical feature is conceptualized as a cooperative “player” within a coalition, synergistically contributing to the model’s ultimate predictive payout. To mathematically evaluate this feature sensitivity, the exact SHAP formulation is expressed as follows:


$$\begin{array}{c} f\left(x\right)=\phi_{\text{0}}+\sum_{i=1}^{M}\phi_{i} \end{array}$$ (12)

Where:

f(x) is the objective function of each variable plus the mean value of the model ϕ_o_

M is the number of features in the model

ϕ_i_ is the SHAP value of feature i

The isolated influence of a specific feature is mathematically determined by evaluating the differential variance in the model’s output as that feature is sequentially introduced into all possible combinatorial subsets of the input space [59]. By computing the weighted average of these marginal contributions across every possible permutation of the remaining variables, the SHAP framework guarantees that the allocation of feature importance is strictly consistent and fortified by robust theoretical axioms [58]. Through this rigorous combinatorial mechanism, SHAP accurately isolates the relative magnitude of influence each input parameter exerts on the predictive outcome, simultaneously elucidating the complex interaction dynamics between the independent variables and the target predictions. Furthermore, beyond merely establishing a macroscopic hierarchy of global feature importance, this advanced methodology uniquely facilitates micro-level interpretability, allowing for the granular deconstruction of individual feature contributions within any specific, localized prediction instance.

The SHAP analysis results of the Weighted Ensemble (GA) model are shown in Fig 13. It can be seen that the Bar Plot (Fig 13a) and Summary Plot (Fig 13b) provide a dynamic hierarchy controlling settlement that is entirely logical with the geotechnical basis. Load (P) and free pile length ($L_{f}$) are identified as the two largest overall influencing factors (with average influence levels of 15.5 mm and 6.8 mm, respectively). Observing Fig 13b, the high-value data points (red) for P and $L_{f}$ converge completely in the positive SHAP region, confirming the absolute covariance: the greater the load and the longer the unembedded pile section, the greater the overall settlement. Conversely, parameters representing the length of the pile ($L_{s}$), tip resistance ($N_{t}$), lateral friction ($N_{sh}$), structural stiffness ($E_{s}$), and diameter (D) exhibit an inverse effect. Piles with high values in these parameters (red dots) mostly generate negative SHAP values (extending to the left), directly reducing the predicted settlement. This clear classification process demonstrates that the algorithm has automatically and perfectly captured the balance between the applied external forces and the geotechnical resistance.

**Fig 13:**
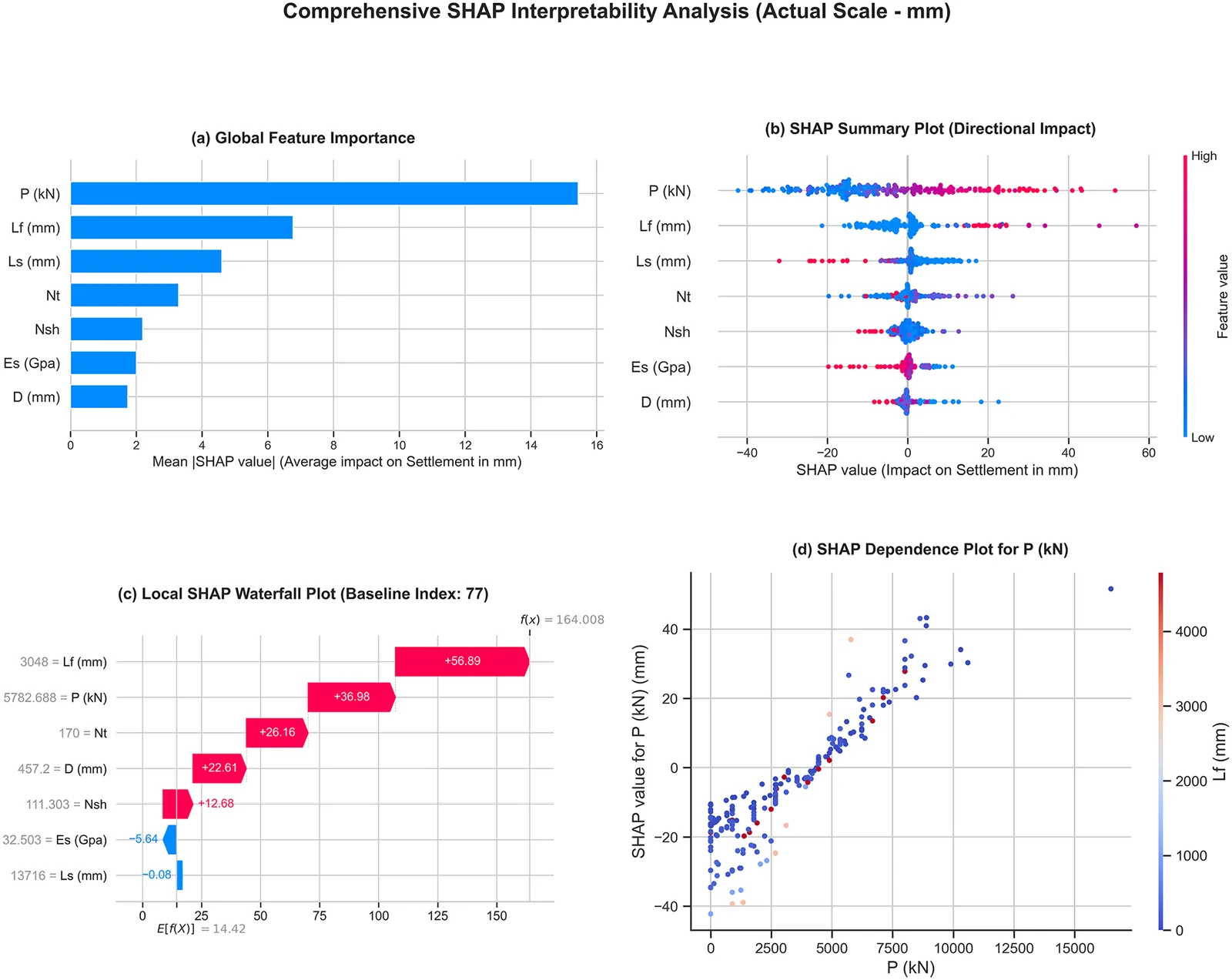
Comprehensive SHAP Interpretability Analysis of the Ensemble Model.

To systematically dismantle skepticism regarding the algorithmic black-box mechanism and decode how the ensemble model reacts to critical geotechnical scenarios, Fig 13c (Waterfall Plot) executes a localized SHAP decomposition for a high-risk test instance (Index: 77). A strategic methodological distinction of this architecture is the prediction of the target variable in a logarithmic space, log(S), effectively controlling heteroscedasticity across extreme loading domains. Consequently, the additive SHAP values depicted in this plot mathematically translate to multiplicative effects on the actual physical settlement. While the baseline expected value of the dataset stands at E[f(x)]= 14.42 mm, the final prediction for Instance 77 is aggressively propelled to an extreme f(x)= 164.008 mm.

A profoundly distinct finding for this specific sample is the critical attribution assigned to the free length ($L_{f}$). The unconfined pile protrusion above the soil stratum ($L_{f}=$ 3048 mm) emerges as the primary aggravating factor, contributing a massive settlement increment of +56.89 mm. Coupled with the extreme applied external load (P= 5782.688 kN), which induces an additional +36.98 mm, the AI implicitly maps, yet significantly transcends, Hooke’s law of linear elasticity. From a purely elastic Hookean perspective, this specific pile segment would merely undergo an axial elastic shortening of approximately 3–5 mm. However, the staggering +56.89 mm penalty allocated by SHAP brilliantly highlights the mechanistic superiority of the ML approach. In this context, the SHAP value does not represent isolated axial deformation; rather, it quantifies the systemic structural degradation. A 3-meter unsupported segment subjected to such extreme axial loading inherently triggers the P-Delta effect (structural buckling and instability), effectively nullifying upper shaft friction and directly transferring critical stress to the lower strata, thereby precipitating a plunging failure. The ML actively penalized the global systemic instability rather than merely computing simplistic elastic compression.

Paradoxically, although the pile was driven into a highly competent soil profile characterized by exceptional geotechnical indices (tip resistance $N_{t}=170$ and shaft friction $N_{sh}=111.303$, denoting an extremely dense/stiff stratum), the model still assigned significant positive SHAP penalties to these parameters (+26.16 mm and +12.68 mm, respectively). This anomaly exposes a profound load-resistance asymmetry mechanism internalized by the algorithm: Under the extreme stress generated by the massive load (P= 5782.688 kN) concentrated on a restricted pile cross-section (D= 457.2 mm), the ultimate bearing capacity of the stiff soil matrix was mobilized beyond its critical threshold, thereby entering a plastic yielding phase. The algorithm correctly evaluated the relative nature of bearing capacity – even an exceptionally competent soil stratum becomes strictly insufficient and directly exacerbates settlement when external forces eclipse its maximum shear strength. Consequently, the limited cross-sectional diameter (D) was severely penalized (+22.61 mm) for providing an inadequate geometrical area to dissipate this catastrophic stress concentration.

The SHAP dependence plot for the variable P (Fig 13d) clearly illustrates the load-displacement (P-S) trajectory of the pile system. At low load ranges (P<4000kN), SHAP has a deeply negative sign, acting as a force pulling the predicted value below the average. When P exceeds 4500 kN, the curve crosses the horizontal axis and accelerates sharply towards the positive region, marking a phase shift to the ultimate resistance mobilization state. It is crucial to note that the selection of the free length ($L_{f}$) as the interaction parameter in the dependence plot was not manually explicitly programmed.

While global feature importance and localized single-instance inspections (such as the baseline high-settlement evaluation in Fig 13c) reveal overarching model sensitivities, relying on a solitary case study risks single-case bias and fails to demonstrate statistical representativeness across diverse geotechnical regimes. To rigorously address this limitation, Fig 14 extends the localized diagnostic framework through a comprehensive multi-group SHAP waterfall decomposition. By capturing four distinct behavioral clusters – namely (a) slender buckling-prone piles, (b) friction-dominated soft soil profiles, (c) linear elastic domains, and (d) extreme load-bearing failure instances – this comparative panel validates that the Weighted Ensemble architecture maintains consistent, physically sound marginal attributions across varying operational boundaries.

**Fig 14:**
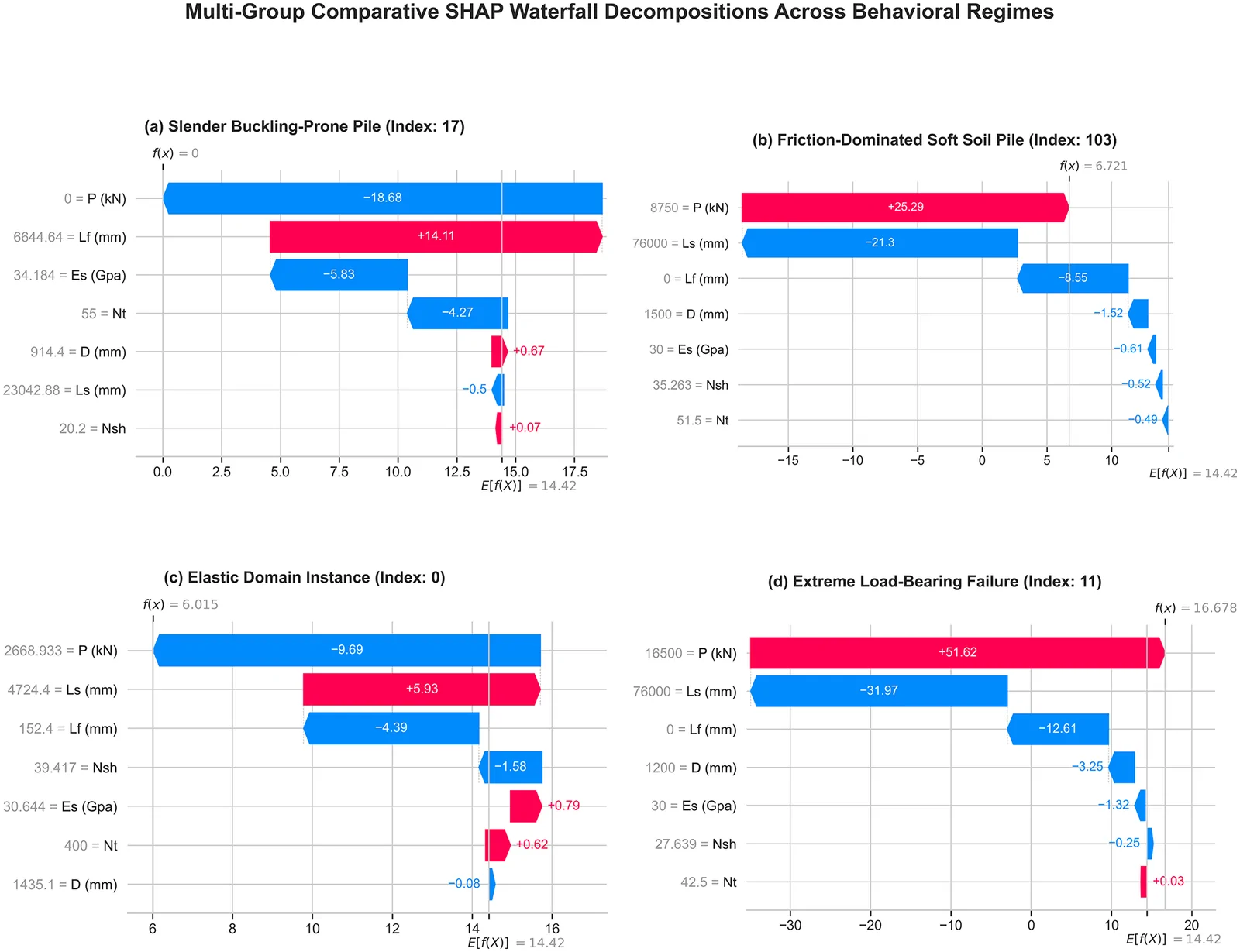
Multi-group comparative SHAP waterfall decompositions across distinct geotechnical behavioral regimes: (a) – Slender buckling-prone pile highlighting unsupported length dominance; (b) – Friction-dominated soft soil pile; (c) – Elastic domain instance showing balanced response; and (d)- Extreme load-bearing failure demonstrating massive axial compression dominance (P).

A rigorous examination of the multi-group waterfall decompositions reveals that the Weighted Ensemble architecture captures distinct, physically consistent behavioral mechanisms across varying geotechnical regimes. Specifically, the slender buckling-prone pile (Index 17) highlights the dominant positive attribution of an extended unsupported length ($L_{f}$ = 6,644.64 mm), confirming the model’s sensitivity to structural instability and flexural effects. In the friction-dominated soft soil profile (Index 103), substantial frictional embedment ($L_{s}$ = 76,000 mm) drives key negative load-transfer resistances, validating the representation of side-friction mechanisms in soft strata. Within the linear elastic domain (Index 0), moderate loading (P = 2,668.933 kN) yields a balanced equilibrium of marginal components, reflecting stable, low-magnitude elastic shortening (f(x) = 6.015 mm). Finally, the extreme load-bearing failure instance (Index 11) demonstrates an overwhelming positive contribution from massive axial compression (P = 16,500 kN), successfully pushing the system into high-nonlinear settlement bounds (f(x) = 16.678 mm) and proving the model’s robustness at data boundaries.

Overall, the SHAP explainer identified the free length ($L_{f}$) as exhibiting a strong mathematical interaction variance with the applied load (P). This algorithmic pattern aligns with the underlying variables of structural elastic deformation ($\Delta S=\frac{P\cdot L_{f}}{E_{s}\cdot A}$), indicating that the Weighted Ensemble architecture successfully reflects the coupling characteristics of elastic compression and geometric nonlinearity without explicit physical programming. Furthermore, multi-group SHAP waterfall decompositions across diverse behavioral clusters – ranging from slender buckling-prone piles to high-load end-bearing failures – validate the consistency and robustness of these attributions, while acknowledging that SHAP values represent marginal contributions rather than absolute structural displacements.

## 5. Conclusions

This study successfully developed and empirically validated an advanced bored pile settlement prediction system predicated on a Weighted Ensemble Learning architecture. By strategically integrating a two-stage hybrid optimization framework (Random Search coupled with a Genetic Algorithm) alongside a SHAP-based interpretability paradigm within a real physical domain, this research not only elevates predictive accuracy but also rigorously resolves the inherent “black-box” dilemma pervasive in geotechnical Artificial Intelligence applications. The core conclusions and primary academic contributions of this study are summarized as follows:

**Superior Predictive Performance and Error Compensation Mechanisms:** The proposed ensemble architecture established an exceptionally high accuracy benchmark on an independent blind test dataset, achieving a coefficient of determination $R^{\text{2}}$ = 0.958, MAE = 0.2322, NRMSE = 5.27%, and RMSE = 0.380 (in the ${log}_{10}$ scale). The RS-GA hybrid optimizer allocated weights with acute mechanistic precision: virtually eliminating the linear model (LR: 3.48%) due to its structural inadequacy, delegating dominant predictive authority to XGBoost (47.88%) to aggressively track highly non-linear failure bounds, while strategically deploying MLP (28.48%) as a continuous spatial smoother and Random Forest (20.16%) as a variance-stabilizing anchor.
**Reconstruction of Continuous and Physically Consistent Load-Settlement (P-S) Trajectories:** Transcending the discrete point interpolation limitations inherent in conventional predictive studies, the ensemble architecture successfully reconstructed the entire continuous P-S trajectory. The integration of the neural network’s (MLP) continuous function approximation capabilities effectively eradicated the step-wise discontinuities systematically associated with pure tree-based models. The proposed framework accurately pinpointed yield inflection zones, flawlessly tracking the complex mechanics of shaft friction degradation and tip resistance mobilization without necessitating any empirical calibration coefficients.
**Transparency of AI Logic and Mechanistic Discovery via XAI:** The Explainable AI (XAI) analysis definitively proved that the machine learning algorithm autonomously internalized fundamental geotechnical mechanics at both macroscopic and microscopic levels. Globally, the SHAP dependence plot identified the free length ($L_{f}$) and applied load (P) as key interacting drivers, demonstrating that the model successfully captures the coupling characteristics of elastic compression and geometric nonlinearity across the dataset. Locally, through the granular deconstruction of a high-risk test instance (Sample Index 77), the study validated the model’s precise “diagnostic” capabilities. The system accurately quantified the structural shortening and localized instability induced by the unconfined pile protrusion, while concurrently recognizing the load-resistance asymmetry when extreme plunging stress overwhelmed even highly competent stiff soil strata. This capability fundamentally elevates the AI from a purely statistical tool into a highly reliable, physically informed expert decision-support system.
**Methodological Limitations and Future Research Trajectories:** Despite achieving rigorous global statistical optimization, purely data-driven models occasionally violate localized physical laws at the dataset boundaries (e.g., the non-physical backward settlement anomaly observed in Pile 36) due to the absence of strict monotonic constraints. Acknowledging this limitation, an inevitable future trajectory is the systematic integration of this hybrid optimization framework into a Physics-Informed Machine Learning (PIML) paradigm. Directly embedding geotechnical mathematical constraints (such as elastoplastic yielding boundaries and strict monotonic conditions) into the loss function will guarantee the model’s absolute compliance with fundamental physical principles across all interpolation spaces. Furthermore, expanding this framework to directly incorporate Monte Carlo reliability analysis will ultimately yield a comprehensive probabilistic foundation design tool for practicing geotechnical engineers.

## Supporting information

S1 Fig Continuous Load-Settlement (P-S) Curves for Piles 13–24. (PNG)

S2 Fig Continuous Load-Settlement (P-S) Curves for Piles 25–36. (PNG)

S3 Fig Continuous Load-Settlement (P-S) Curves for Piles 37–51. (PNG)

S1 File Static Load Test (SLT) Database. This file contains the complete dataset encompassing 542 discrete load-settlement data points used for model training and testing.
(CSV)

S2 File Optimization and Modeling Source Code. Python scripts containing the Hybrid RS-GA Weighted Ensemble architecture to facilitate full reproducibility.
(PY)
